# Supplementary material for: Single-cell signaling network profiling during redox stress reveals dynamic redox regulation in immune cells
Source: Nat Commun. 2025 Jul 1;16:5600. doi: 10.1038/s41467-025-60727-z (PMC12215076; doi:10.1038/s41467-025-60727-z)
Supplement: Supplementary file 1 — Supplementary Information [file 41467_2025_60727_MOESM1_ESM.pdf]

**Supplementary Information**

**Title:**

Single-cell signaling network profiling during redox stress reveals dynamic redox regulation in immune cells

**First Author:** Yi-Chuan Wang

**Corresponding Author:** Shih-Yu Chen

sychen@ibms.sinica.edu.tw

**a**

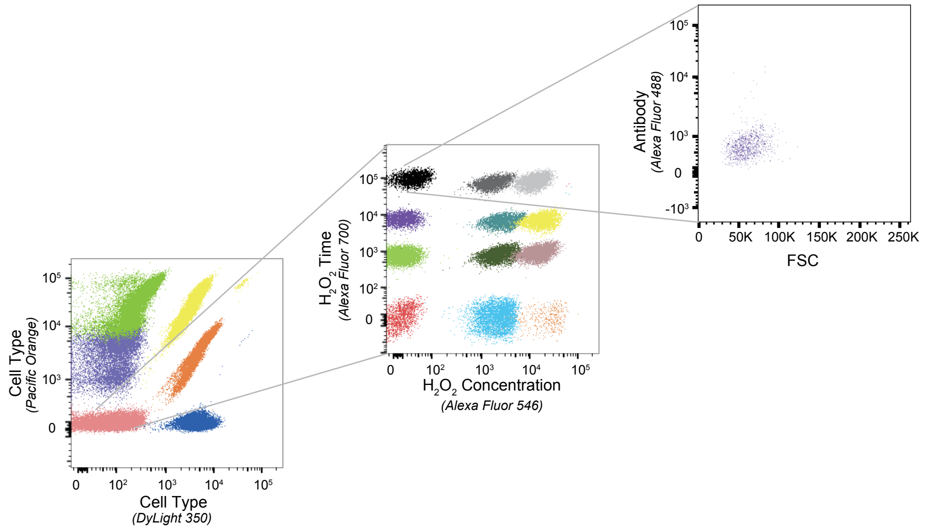


**b**

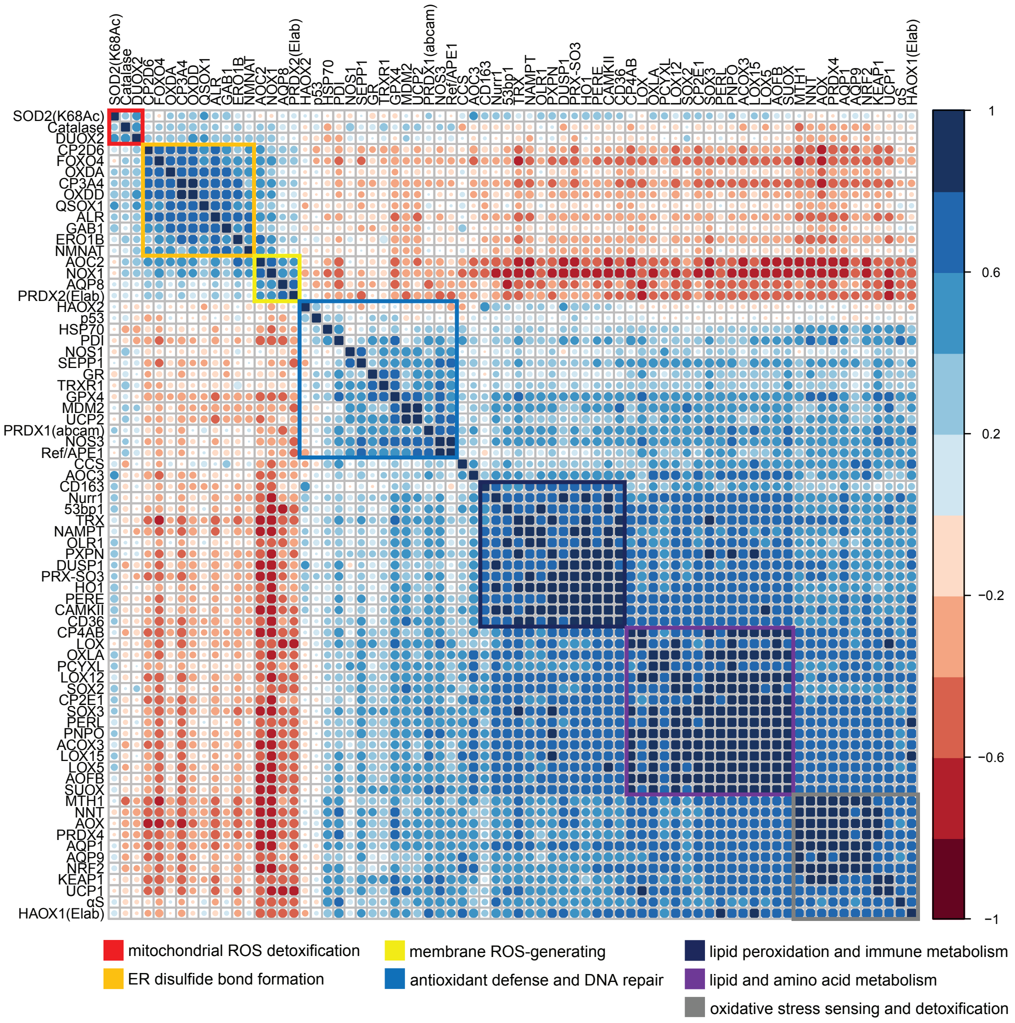


**Supplementary Figure 1| Selection of antibodies for SN-ROP panel. a,** Fluorescence from indicated dyes in cells from six lines (distinguished by color) exposed to different concentrations (0, 10, and 100 µM) of $H_2O_2$ for 0, 0.5, 4, and 48 hours. The cells were stained using amine-reactive fluorescent dyes Pacific Orange and DyLight 350 at concentrations of 0, 0.1, or 1 µg/ml. Additionally, the cells were labeled with Alexa Fluor 546 at concentrations of 0, 0.1, or 1 µg/ml and Alexa Fluor 700 at concentrations of 0, 0.01, 0.07, or 1 µg/ml. The candidate antibodies were labeled using Alexa Fluor 488. **b,** Heatmap of Spearman correlation coefficients between across six immune cell types with data from 12 different $H_2O_2$ conditions. All values were transformed using the ASINH function. Antibodies were grouped into seven modules based on their correlation patterns: red, mitochondrial ROS detoxification; orange, endoplasmic reticulum disulfide bond formation; yellow, membrane ROS-generating; blue, antioxidant defense and DNA repair; dark blue, lipid peroxidation and immune metabolism; purple, lipid and amino acid metabolism; and gray, oxidative stress sensing and detoxification. The color scale ranges from red (negative correlation) to blue (positive correlation).

**a**

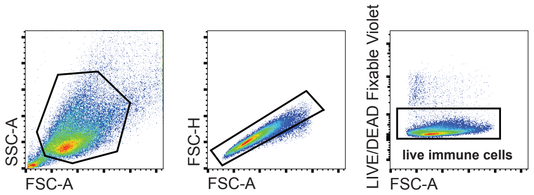

SSC-A
FSC-A
FSC-H
FSC-A
LIVE/DEAD Fixable Violet
live immune cells
FSC-A


**b**

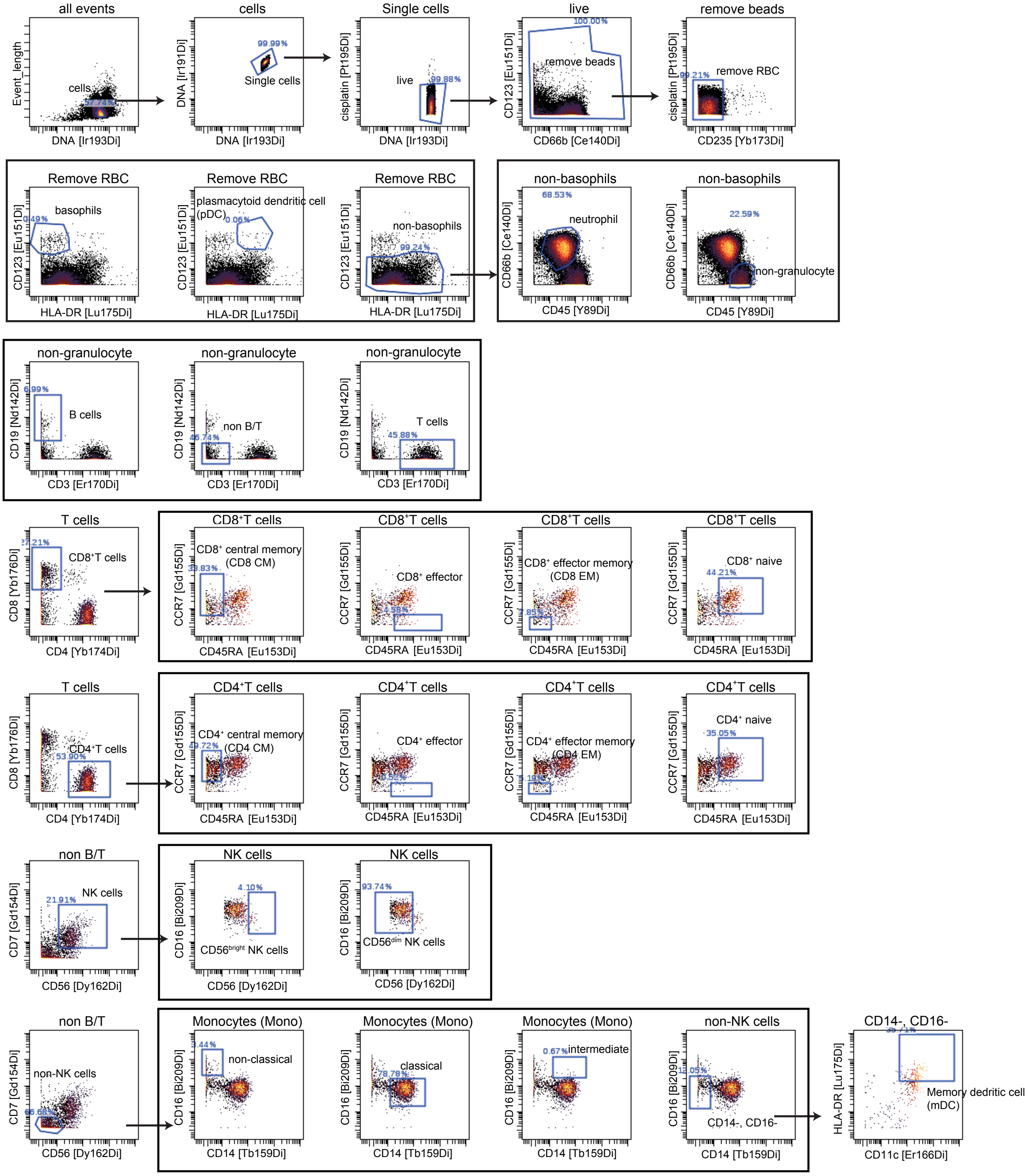

all events
Event_length
cells
DNA [Ir193Di]
cells
99.99%
DNA [Ir191Di]
Single cells
DNA [Ir193Di]
Single cells
cisplatin [Pt195Di]
live
99.88%
DNA [Ir193Di]
live
100.00%
CD123 [Eu151Di]
remove beads
CD66b [Ce140Di]
remove beads
99.21%
cisplatin [Pt195Di]
remove RBC
CD235 [Yb173Di]
Remove RBC
0.49%
basophils
CD123 [Eu151Di]
HLA-DR [Lu175Di]
Remove RBC
plasmacytoid dendritic cell
(pDC)
0.06%
CD123 [Eu151Di]
HLA-DR [Lu175Di]
Remove RBC
non-basophils
99.24%
CD123 [Eu151Di]
HLA-DR [Lu175Di]
non-basophils
68.53%
neutrophil
CD66b [Ce140Di]
CD45 [Y89Di]
non-basophils
22.59%
non-granulocyte
CD66b [Ce140Di]
CD45 [Y89Di]
non-granulocyte
6.99%
B cells
CD19 [Nd142Di]
CD3 [Er170Di]
non-granulocyte
non B/T
45.74%
CD19 [Nd142Di]
CD3 [Er170Di]
non-granulocyte
T cells
45.88%
CD19 [Nd142Di]
CD3 [Er170Di]
T cells
CD8⁺T cells
CD8 [Yb176Di]
CD4 [Yb174Di]
CD8⁺T cells
CD8⁺ central memory
(CD8 CM)
38.83%
CCR7 [Gd155Di]
CD45RA [Eu153Di]
CD8⁺T cells
CD8⁺ effector
4.58%
CCR7 [Gd155Di]
CD45RA [Eu153Di]
CD8⁺T cells
CD8⁺ effector memory
(CD8 EM)
CCR7 [Gd155Di]
CD45RA [Eu153Di]
CD8⁺T cells
CD8⁺ naive
44.21%
CCR7 [Gd155Di]
CD45RA [Eu153Di]
T cells
CD4⁺T cells
53.90%
CD8 [Yb176Di]
CD4 [Yb174Di]
CD4⁺T cells
CD4⁺ central memory
(CD4 CM)
CCR7 [Gd155Di]
CD45RA [Eu153Di]
CD4⁺T cells
CD4⁺ effector
CCR7 [Gd155Di]
CD45RA [Eu153Di]
CD4⁺T cells
CD4⁺ effector memory
(CD4 EM)
CCR7 [Gd155Di]
CD45RA [Eu153Di]
CD4⁺T cells
CD4⁺ naive
35.05%
CCR7 [Gd155Di]
CD45RA [Eu153Di]
non B/T
NK cells
21.91%
CD7 [Gd154Di]
CD56 [Dy162Di]
NK cells
4.10%
CD56bright NK cells
CD16 [Bi209Di]
CD56 [Dy162Di]
NK cells
93.74%
CD56dim NK cells
CD16 [Bi209Di]
CD56 [Dy162Di]
non B/T
non-NK cells
CD7 [Gd154Di]
CD56 [Dy162Di]
Monocytes (Mono)
non-classical
CD16 [Bi209Di]
CD14 [Tb159Di]
Monocytes (Mono)
classical
78.78%
CD16 [Bi209Di]
CD14 [Tb159Di]
Monocytes (Mono)
0.67%
intermediate
CD16 [Bi209Di]
CD14 [Tb159Di]
non-NK cells
CD14-, CD16-
CD16 [Bi209Di]
CD14 [Tb159Di]
CD14-, CD16-
HLA-DR [Lu175Di]
Memory dedritic cell
(mDC)
CD11c [Er166Di]

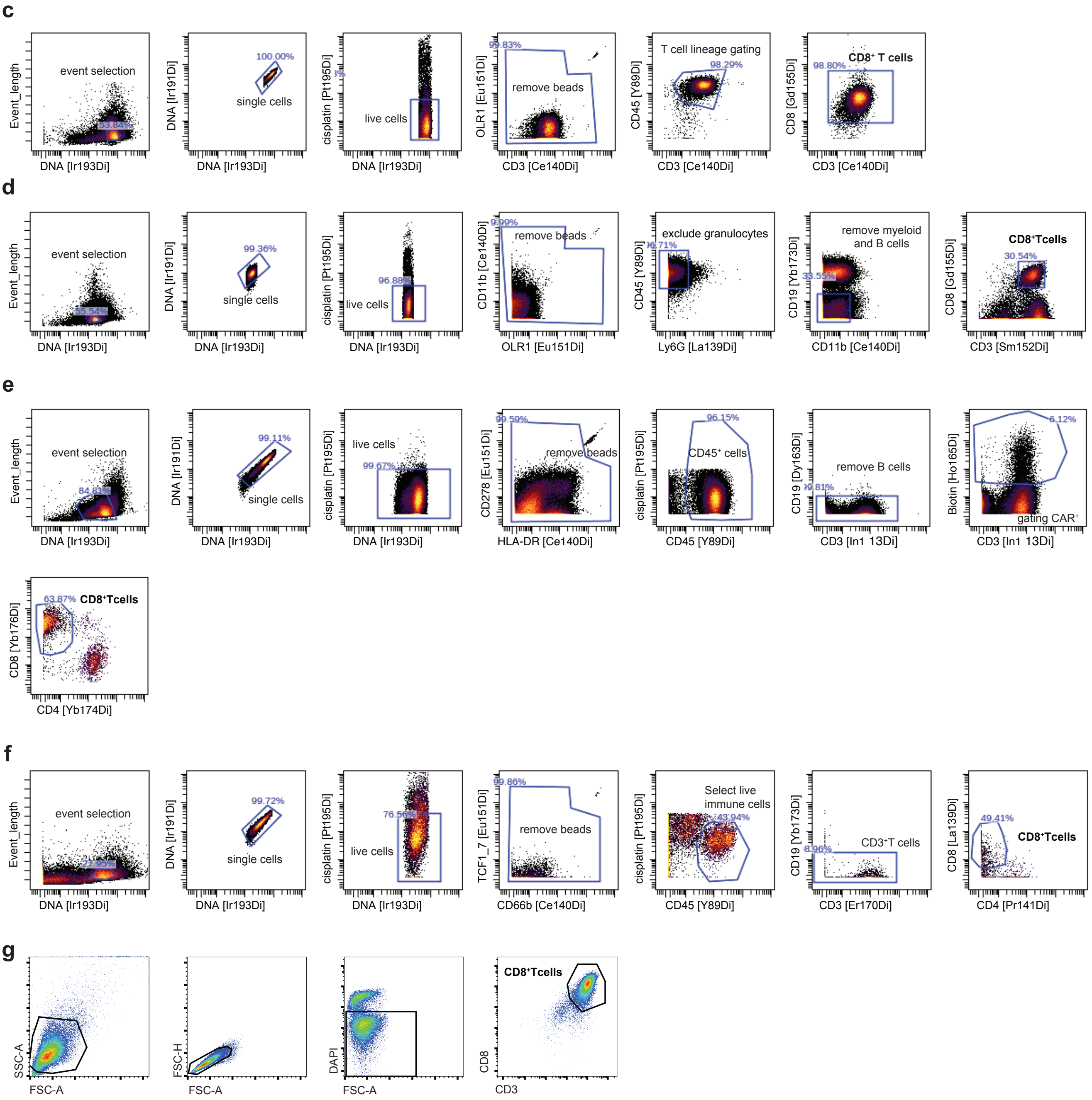


**Supplementary Figure 2| Gating strategies for flow cytometry and CyTOF analysis. a,** Flow cytometry gating strategy for screening six immune cell lines (Raw264.7, SH-SY5Y, HL-1, SM826, HUVEC, and Jurkat) for SN-ROP panel validation. **b,** CyTOF gating strategy for 18 immune cell populations from human whole blood samples. Events were gated on DNA (Ir193Di) and event length, followed by exclusion of doublets and cisplatin⁺ dead cells. Lineage markers were used to identify cell types in healthy donors and hemodialysis patients. **c,** CyTOF gating of OT-I CD8⁺ T cells cultured under normoxia, hypoxia, or antioxidant (N-AC) treatment for SN-ROP profiling. Cells were gated on live, singlet, cisplatin⁺ CD8⁺ T cells. **d,** CyTOF gating strategy for CD8⁺ tumor-infiltrating lymphocytes (TILs) from MC38 tumor–bearing mice and splenic CD8⁺ T cells from LCMV-infected mice. Granulocytes, B cells, and myeloid cells were excluded using Ly6G, CD19, and CD11b. **e,** CyTOF gating strategy for identifying CAR⁺ CD8⁺ T cells in peripheral blood from CAR-T patients. Cells were stained with CD19 CAR Detection Reagent (Biotin), followed by anti-biotin-165Ho. Gating was performed on cisplatin⁺ CD3⁺CD8⁺ T cells positive for biotin. f, CyTOF gating of liver-resident immune cells from hepatocellular carcinoma (HCC) tumors and paired adjacent tissues. Live singlets were gated on CD45⁺ cells after exclusion of debris and beads. CD3⁺CD8⁺ T cells were then identified. **g,** Flow cytometry gating of OT-I CD8⁺ T cells treated with APX2009, N-AC, or both. After PMA/ionomycin restimulation, viable CD3⁺CD8⁺ cells were gated for cytokine expression analysis.

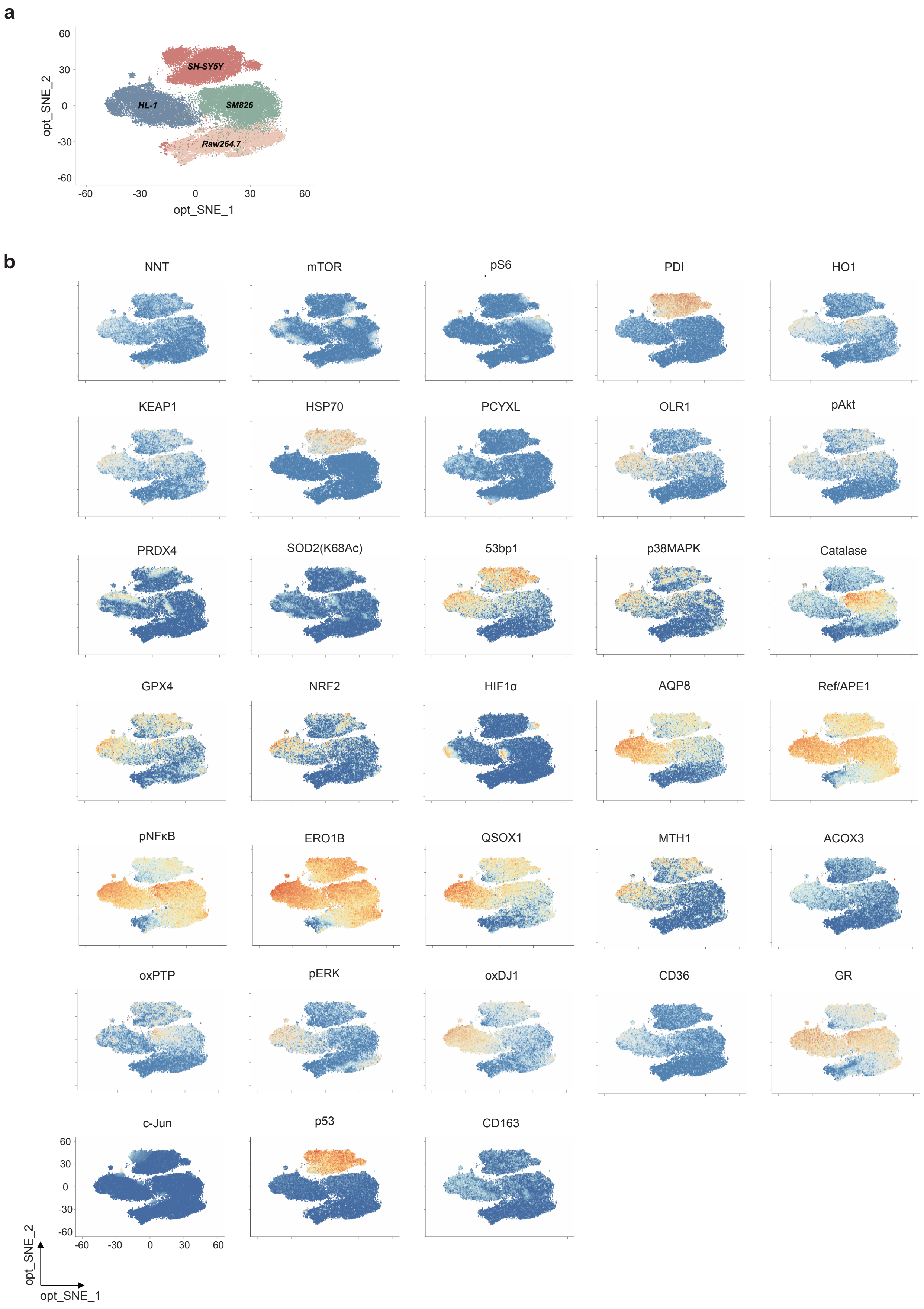


**Supplementary Figure 3| OptSNE analysis of cell line distribution and SN-ROP marker expression. a,** OptSNE plot showing the distinct clustering patterns of four different cell lines: HL-1 (blue), Raw264.7 (pink), SH-SY5Y (hot pink), and SM826 (green). Each dot represents a single cell, with colors corresponding to the cell line. **b,** OptSNE plot illustrating the expression levels of SN-ROP markers across the four cell lines. The intensity of feature expression is mapped onto the plot, highlighting the variation in redox-related protein expression within and between the cell lines.

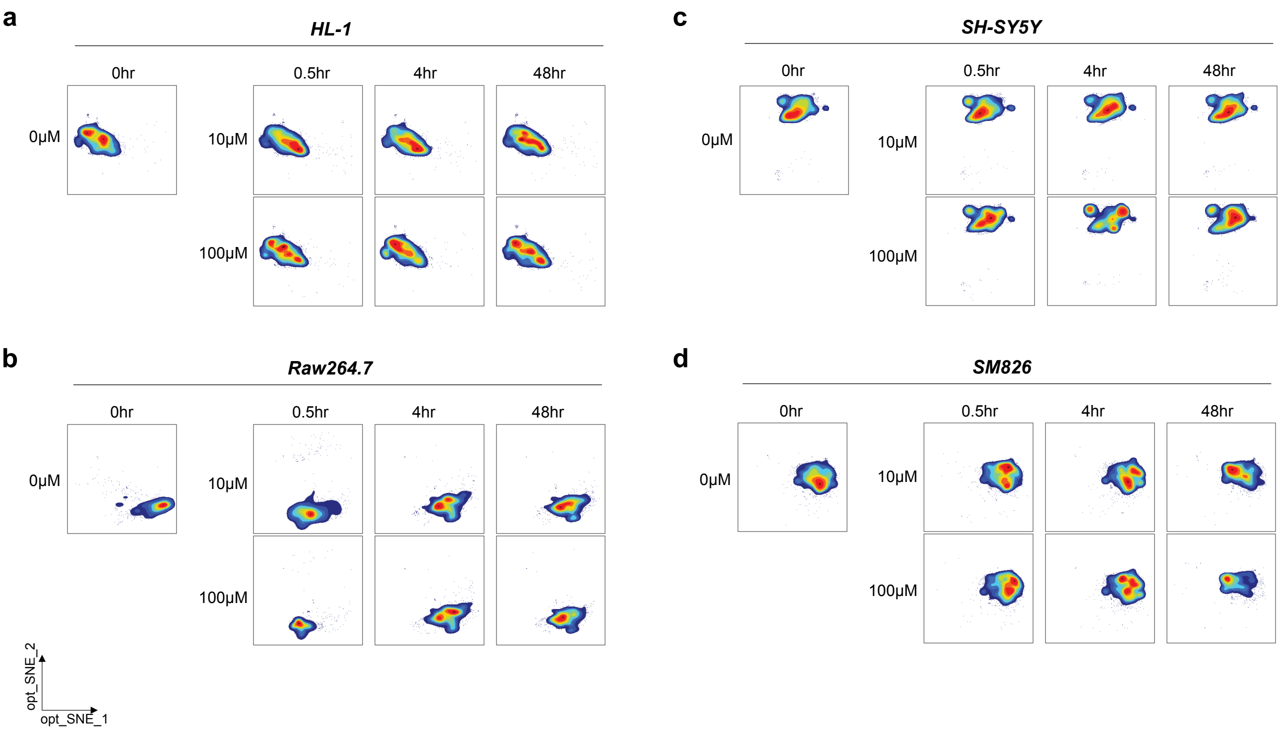


**Supplementary Figure 4| Time courses of responses to $H_2O_2$ for four cell lines.** Contour plots illustrating the responses to $H_2O_2$ of four cell lines: **a,** HL-1, **b,** Raw264.7, **c,** SH-SY5Y, and **d,** SM826. The plots show untreated cells (0 hours, 0 μM $H_2O_2$) and the effects of $H_2O_2$ treatment at various time points (0.5, 4, and 48 hours) and concentrations (10 μM and 100 μM). Each contour represents changes in cell response over time.

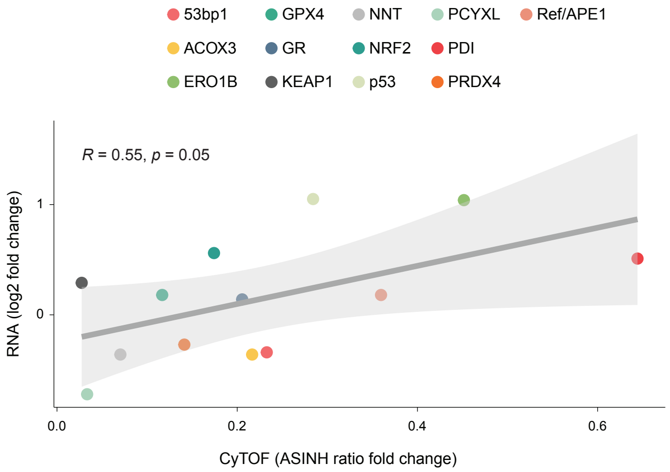


**Supplementary Figure 5| Comparison of SN-ROP to RNA-seq and CyTOF results in Jurkat cells.** Scatter plot comparing the fold changes of 13 SN-ROP markers measured by RNA-seq (log2 values) and CyTOF (ASINH values) in Jurkat cells [20]. Fold changes were calculated as the difference between $H_2O_2$-treated cells (48 hours, 100 μM) and untreated cells (0 hours, 0 μM). Each dot corresponds to a SN-ROP marker with a Pearson correlation coefficient of $R = 0.55$ and a $P$ value of 0.05.

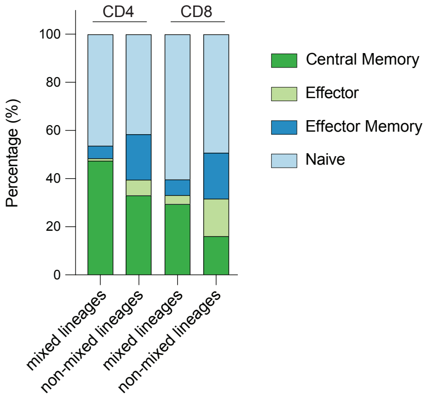


**Supplementary Figure 6| Distribution of T cell subtypes in mixed and non-mixed lineages.** Percentages of $CD4^+$ and $CD8^+$ T cell subtypes in mixed and non-mixed lineages. Dark green represents central memory T cells, light green indicates effector T cells, dark blue denotes effector memory T cells, and light blue signifies naive T cells. The gating for mixed lineages, located next to basophils in the Fig. 1f UMAP plot, includes these cells, whereas the non-mixed lineages consist of T cells that exclude mixed lineages.

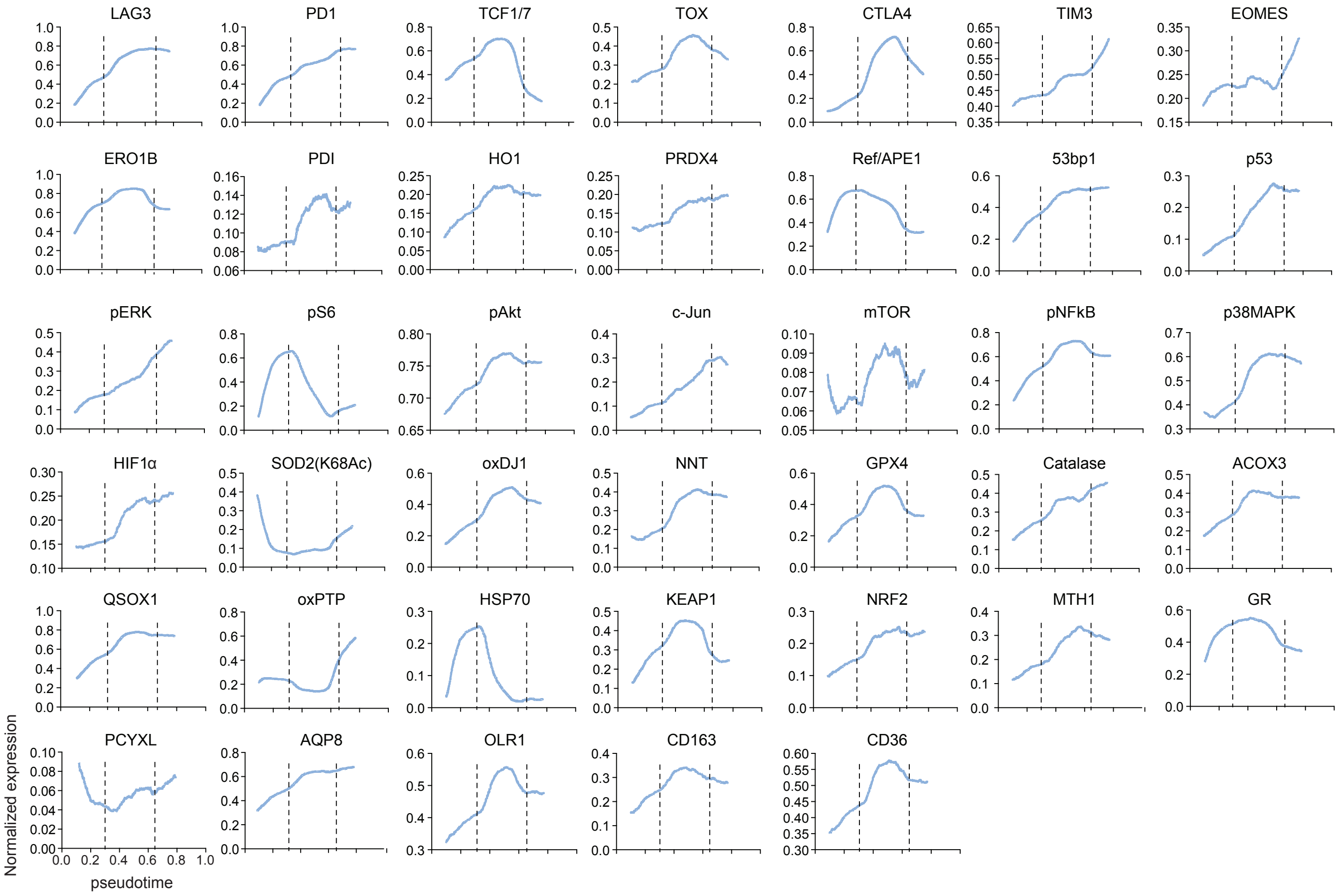


**Supplementary Figure 7| SN-ROP analysis reveals redox network remodeling in CD8⁺ T cells across pseudotime.** Pseudotime line chart plot of the 99th percentile normalized SN-ROP values smoothed using a window size of 1000. The important inflection time points are denoted by vertical dashed lines (*n*=3 independent samples, shown are data for one representative sample).

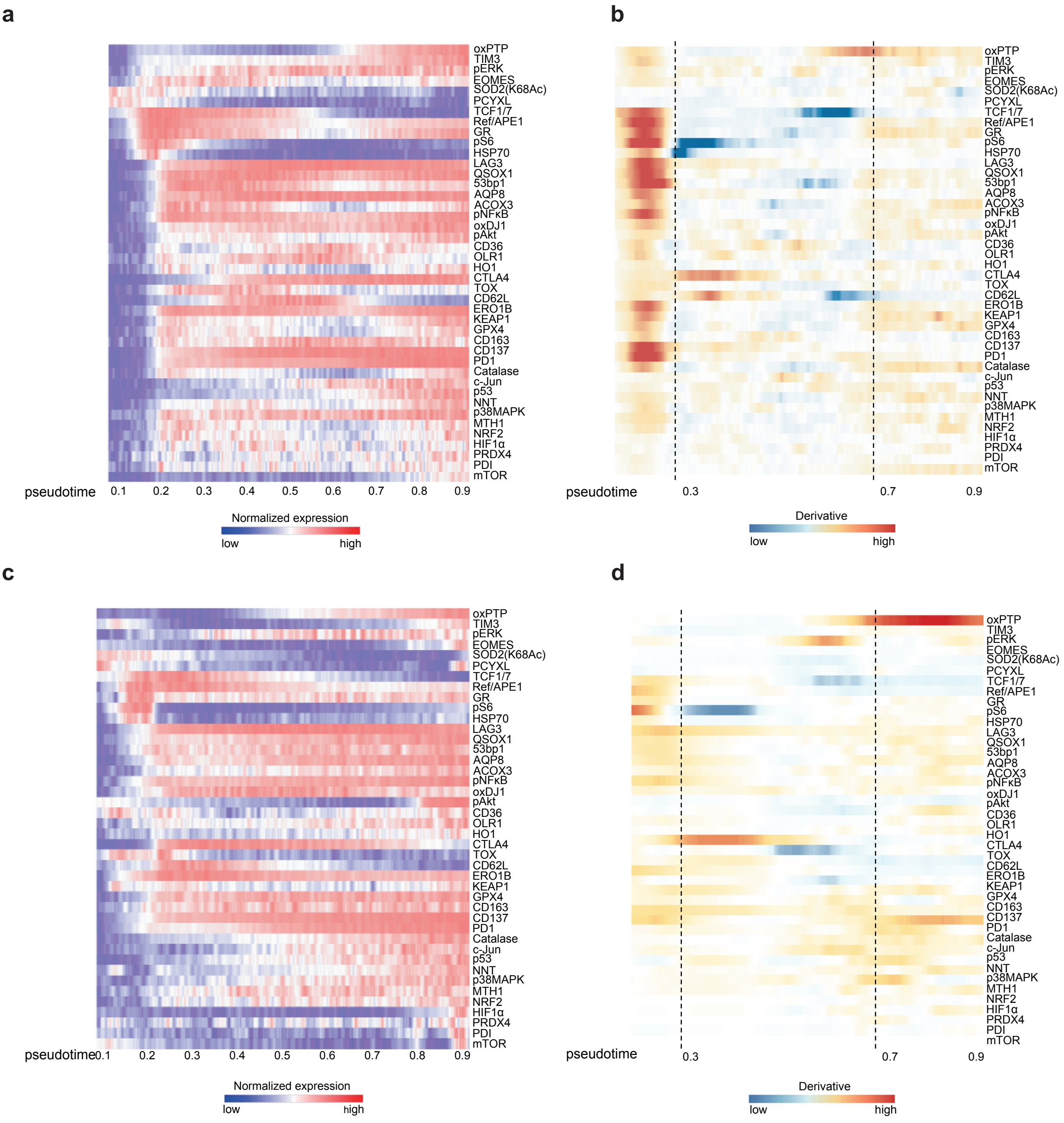


**Supplementary Figure 8| Pseudotime analysis of protein expression in CD8⁺ T cells. a and c,** Pseudotime values calculated using the SCORPIUS package plotted in a heatmap along with the 99th percentile normalized data, which was smoothed using a window size of 100. **b and d,** Slope (first derivative) heatmap of protein expression across pseudotime. The vertical dashed lines indicate significant inflection points (*n*=3 independent samples). Panels a and c show data from two samples.

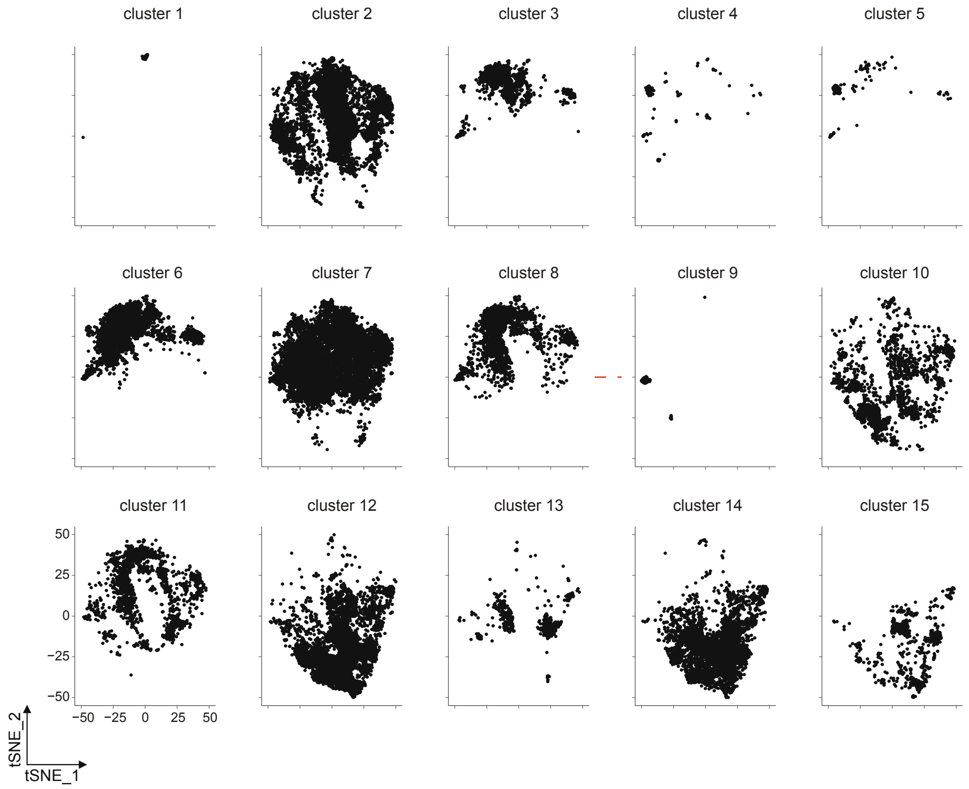


**Supplementary Figure 9| tSNE plot of CAR-positive T cells clustered by redox profiles.** tSNE plot of the distribution of CAR-positive T cells across 15 clusters identified using FlowSOM based on SN-ROP markers.

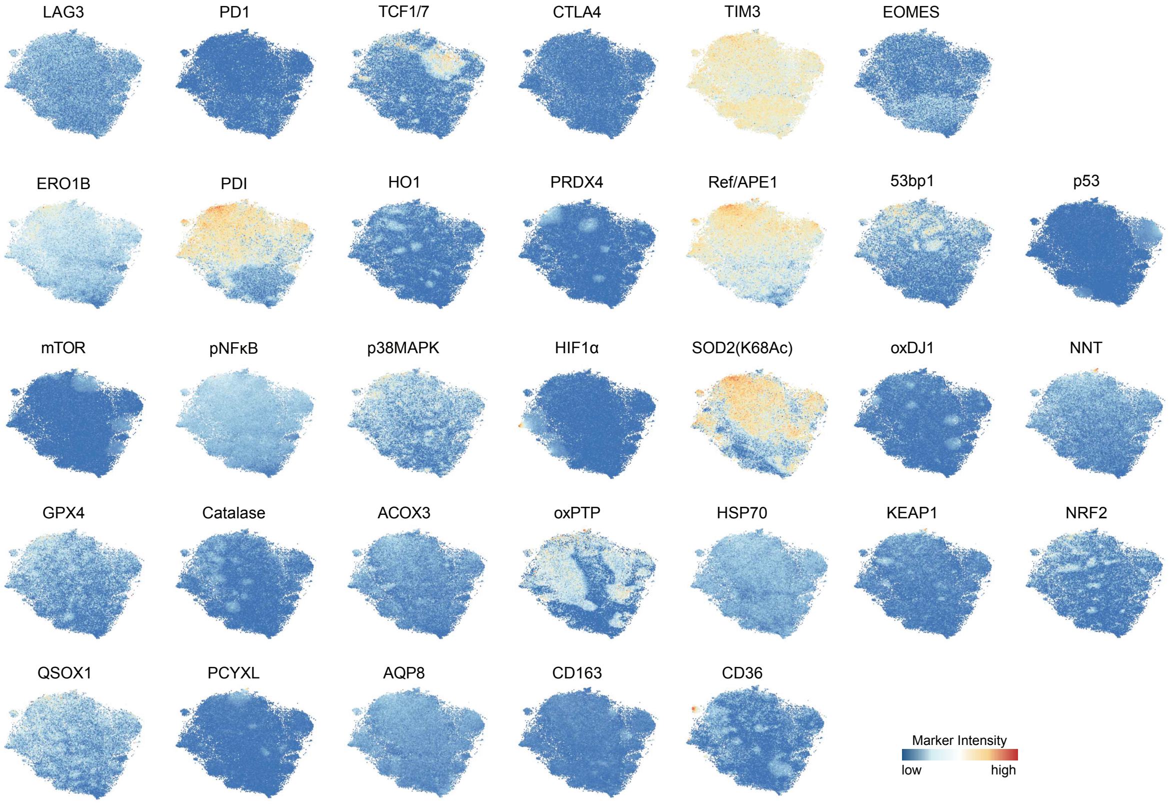


**Supplementary Figure 10| tSNE plots of CD8⁺ CAR-T cells highlight SN-ROP and exhaustion marker expression.** Colors represent the ASINH-transformed expression levels of each SN-ROP marker and exhaustion marker.

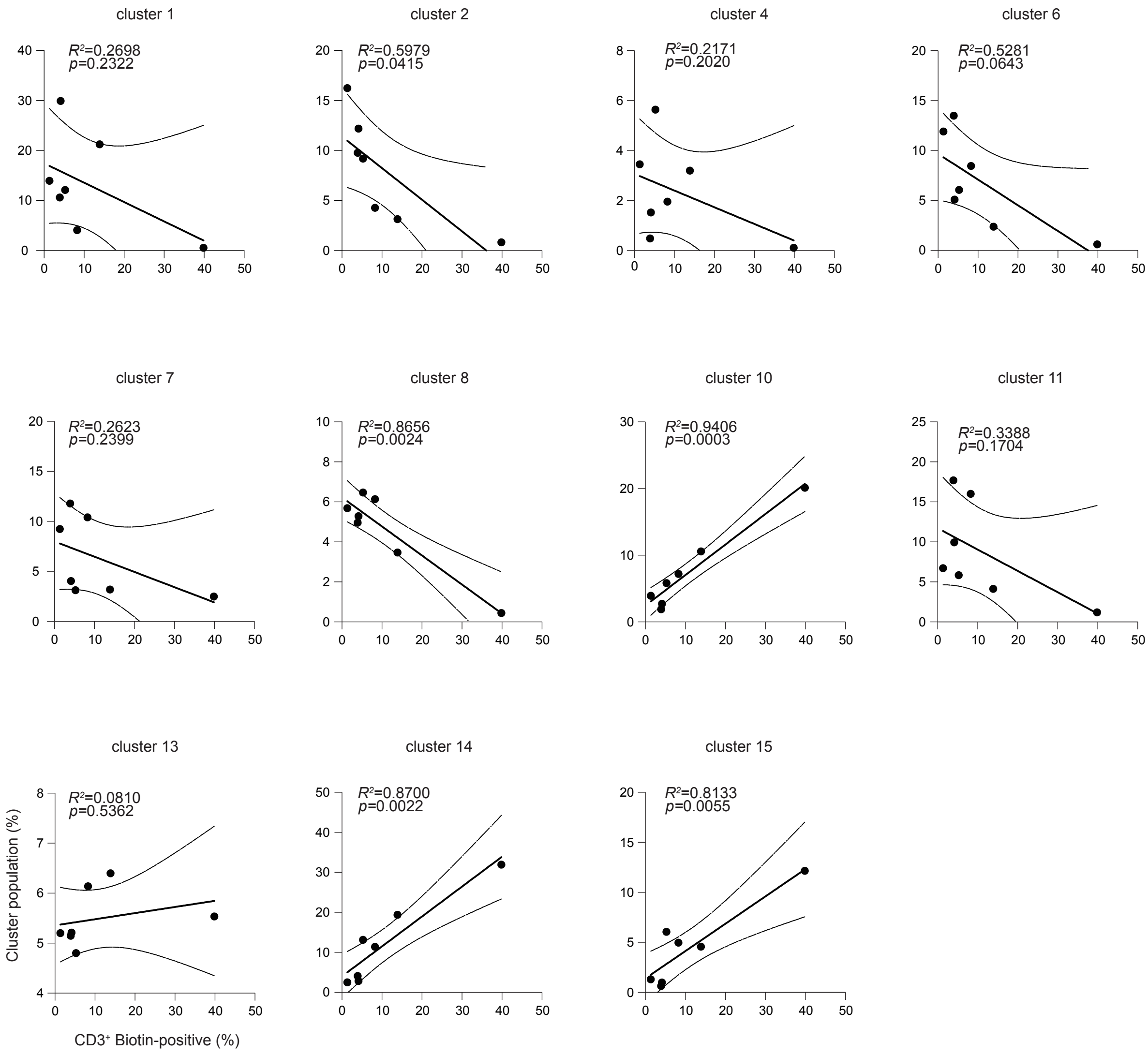


**Supplementary Figure 11| Correlation analyses between SN-ROP-based cell clustering and long-term CAR-T persistence.** Percentages of cells in indicated clusters at 28 days post CAR-T administration were correlated with the percentage of CAR-T cells at day 90. Each dot represents an individual CAR-T patient. The solid line indicates the fitted linear regression, and the flanking lines represent the 95% confidence interval. Pearson correlation coefficients ($R^2$) and exact two-sided $P$ values are shown on each plot. Clusters 3, 5, 9, and 12 are shown in Fig. 4.

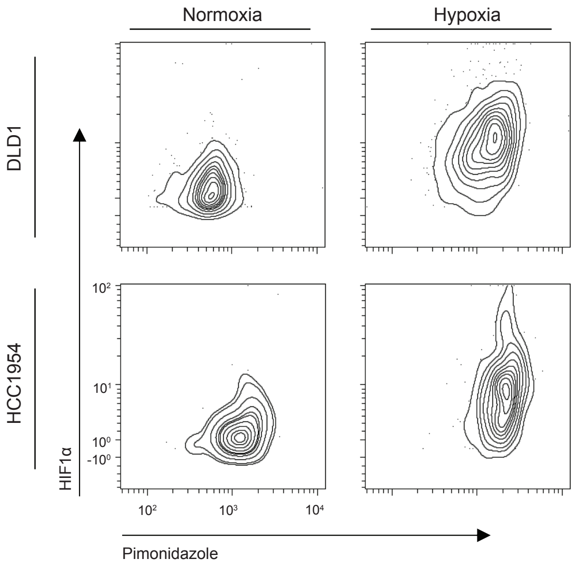


**Supplementary Figure 12| Pimonidazole staining and HIF1α expression in DLD1 and HCC1954 cells.** Biaxial contour plots of pimonidazole staining and HIF1α intensities in two cell lines, DLD1 and HCC1954, cultured under normoxic or hypoxic conditions for 4 hours.

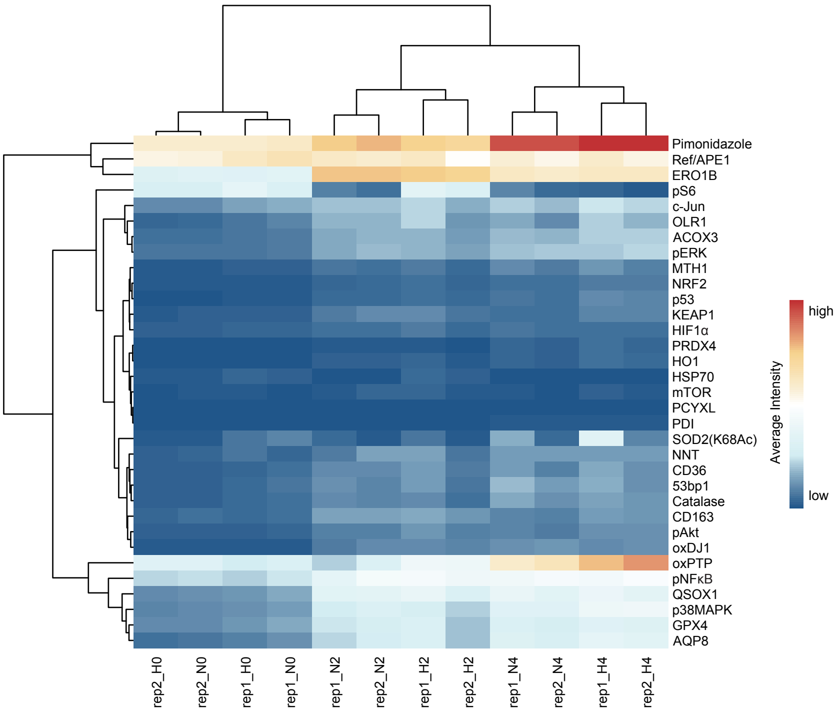


**Supplementary Figure 13| Two independent SN-ROP analyses of activated CD8$^+$ T cells from OT-1 mice cultured under normoxic or hypoxic conditions.** Heatmap of the ASINH transformed levels of pimonidazole staining and SN-ROP marker expression in CD8$^+$ T cells at day 0, day 2, and day 4 under normoxic (N0, N2, N4) or hypoxic (H0, H2, H4) conditions. Data from two independent experiments (rep1 and rep2) are shown.

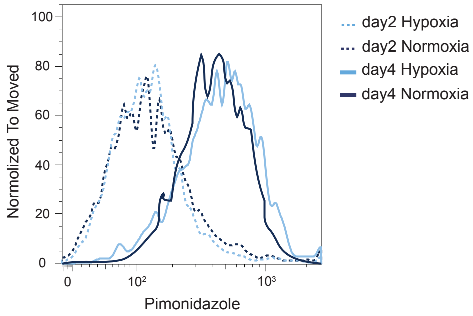


**Supplementary Figure 14| Histogram of pimonidazole staining under hypoxia and normoxia conditions.** Histogram of pimonidazole staining intensities in T cells from OT-1 mice at day 2 under hypoxia (dashed light blue line), day 2 under normoxia (dashed dark blue line), day 4 under normoxia (solid light blue line), and day 4 under hypoxia (solid dark blue line). Data from one of two replicates are shown.

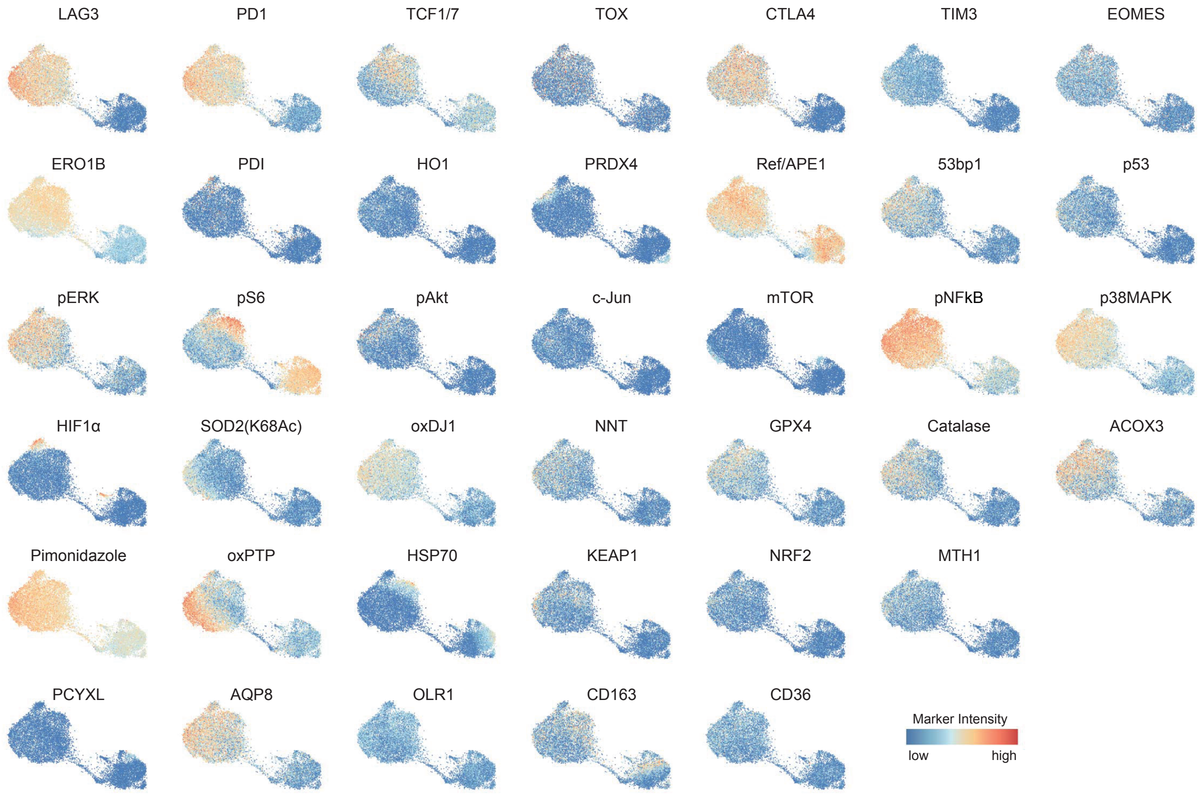


**Supplementary Figure 15| SN-ROP of CD8⁺ T cells from OT-1 mice under normal and hypoxic condition visualized *via* UMAP.** UMAP plot of activation states of the CD8⁺ T cells from the spleens of OT-I mice at days 0, 2, and 4. The visualization incorporates 2000 cells sampled from one of the triplicate experiments. Colors are based on ASINH transformed expression of the each SN-ROP marker and exhaustion marker.

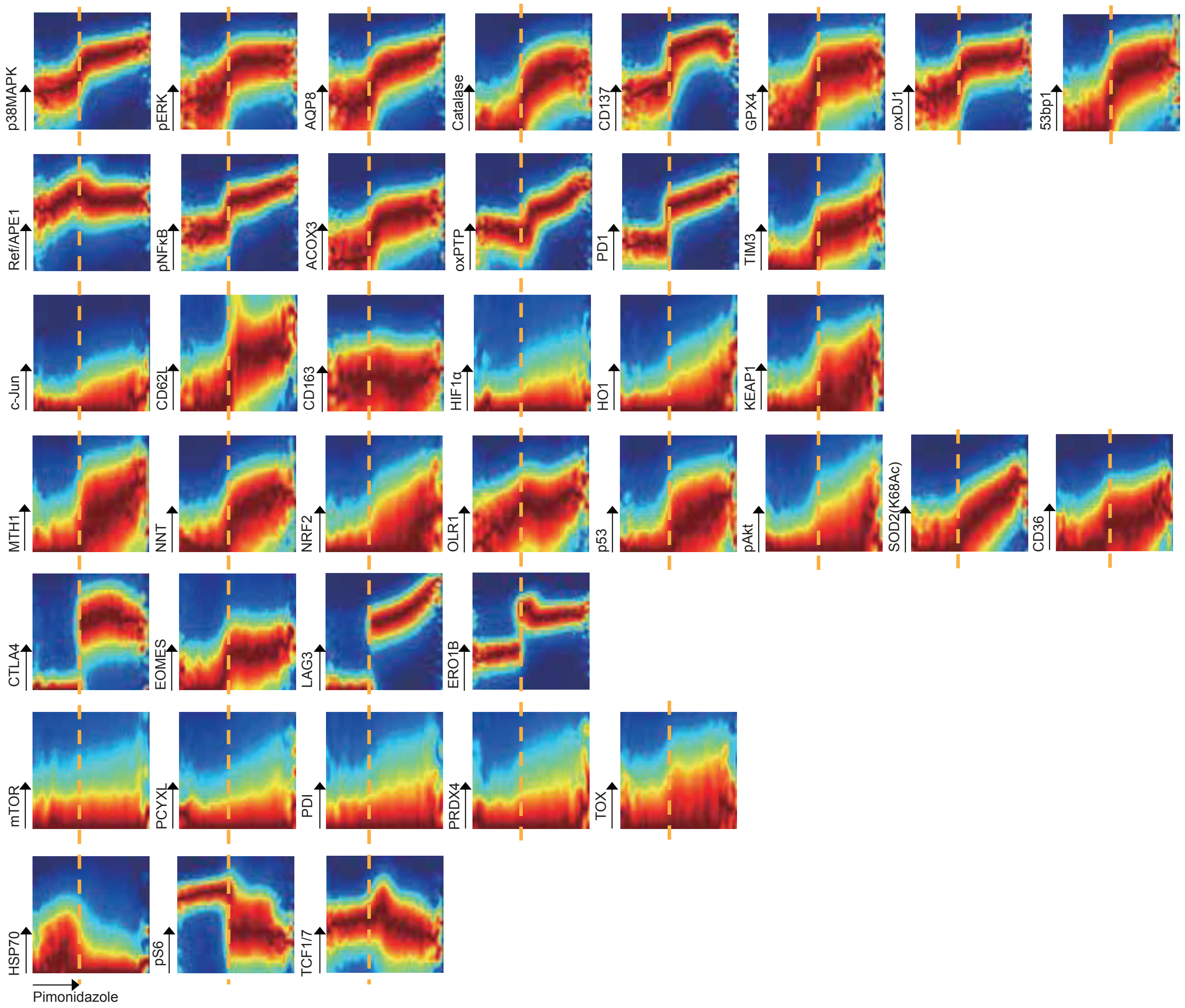


**Supplementary Figure 16| SN-ROP analysis of CD8⁺ T cells from OT-1 mice under normal and hypoxia conditions visualized *via* DREVI plots.** DREVI plots of the distributions of densities of SN-ROP markers in CD8⁺ T cells form OT-1 mice under hypoxic conditions (observed by pimonidazole). Dark red color highlights areas with higher density in the specific slice. The orange dashed line signifies the coordinated transition hypoxic time point.

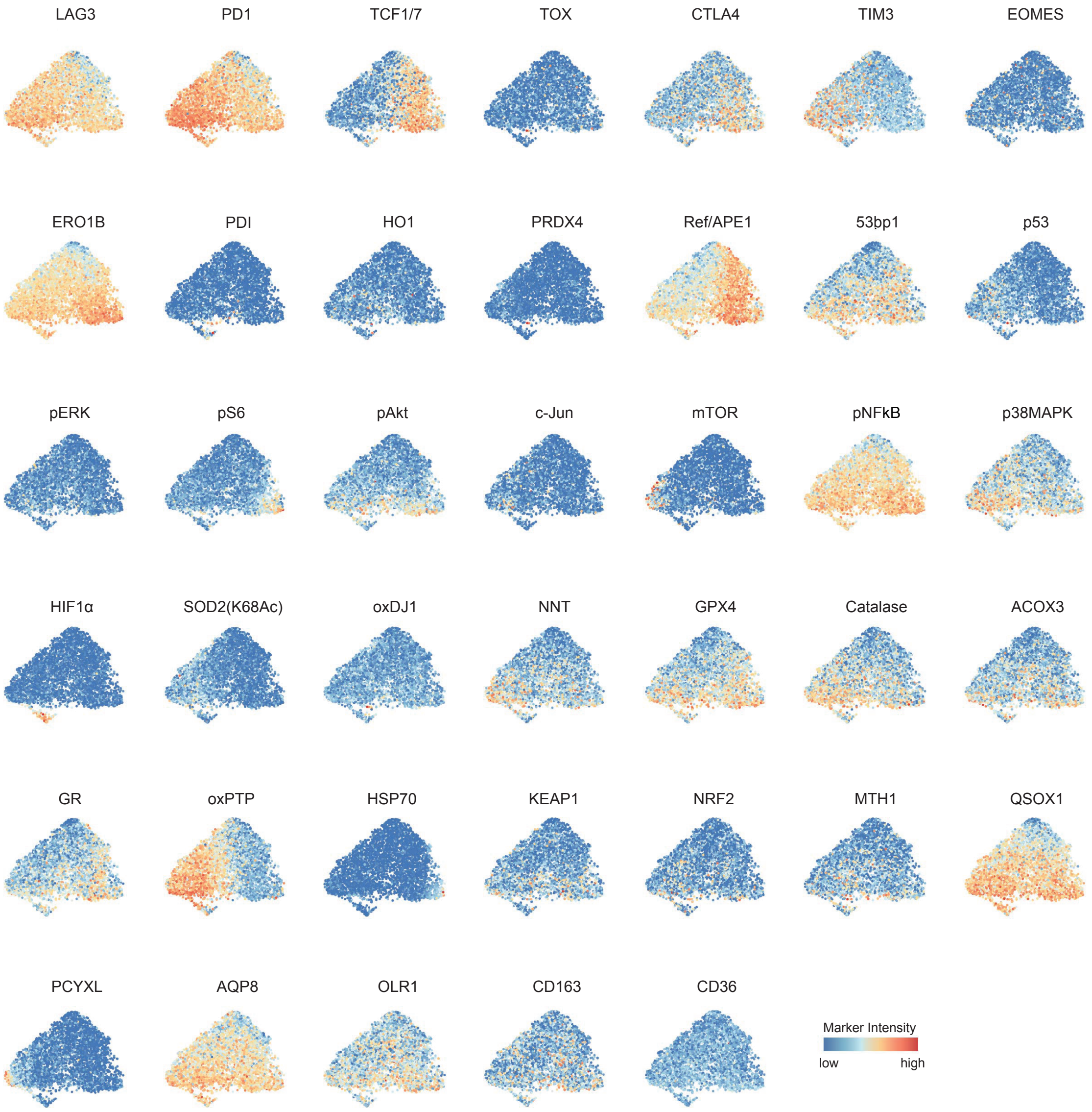


**Supplementary Figure 17| SN-ROP analysis of CD8⁺ T cells from OT-1 mice with or without N-AC treatment visualized *via* UMAP.** Representative UMAP plots showing expression of SN-ROP markers in T cells from OT-1 mice with and without antioxidant N-AC treatment. The visualization incorporates data from 2000 cells sampled from one of the triplicate experiments. Colors are based on ASINH transformed expression of the each SN-ROP marker and exhaustion marker.

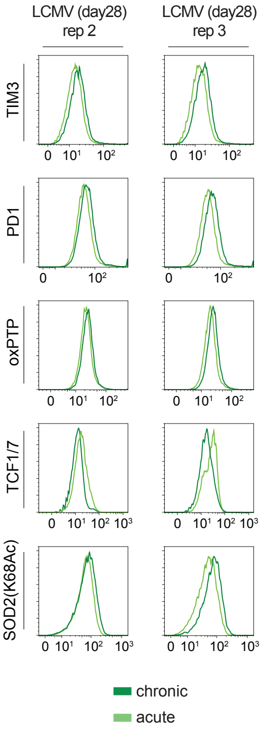


**Supplementary Figure 18| Expression of immune checkpoint inhibitors and SN-ROP markers in LCMV models.** Histogram of mean fluorescence intensities for immune checkpoint inhibitors and SN-ROP markers in LCMV acute (light green) and chronic (dark green) models at day 28 (*n*=3).

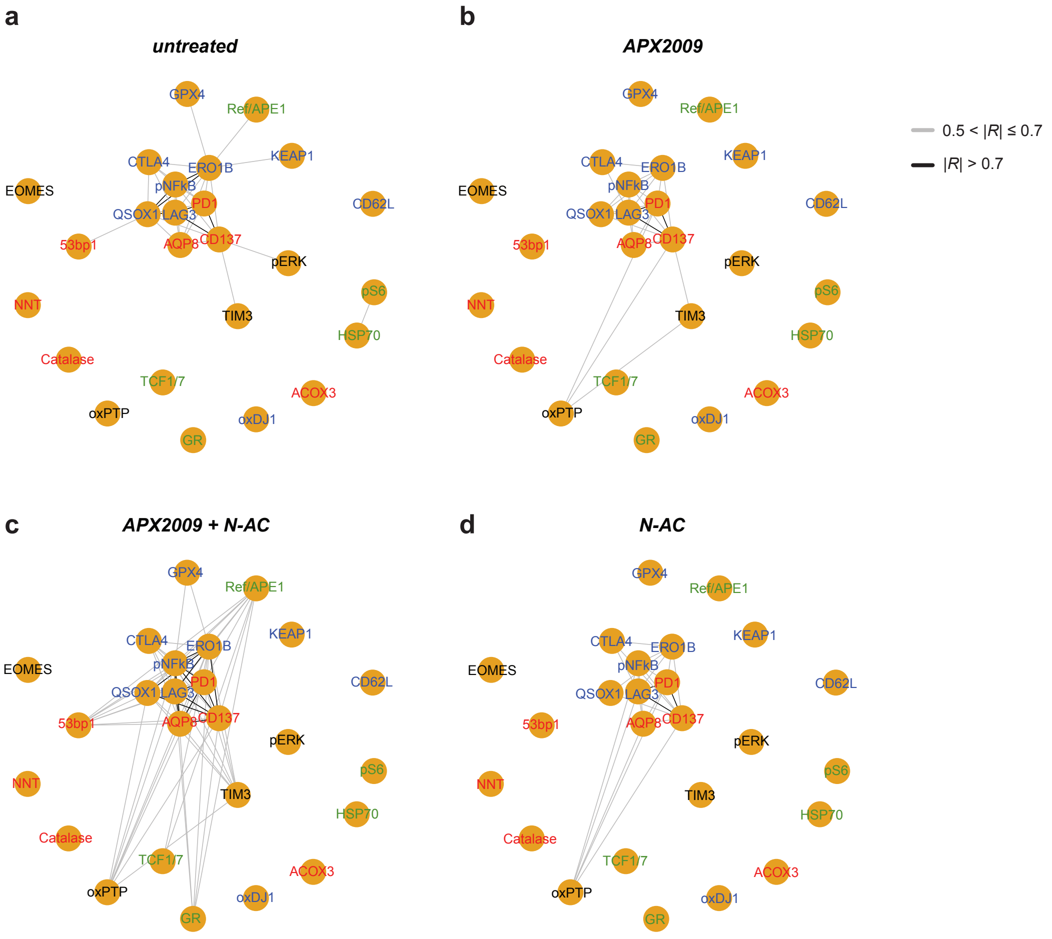


**Supplementary Figure 19| Functional network of features by roles and correlation strengths.** Plots of relationships between features in T cells from OT-1 mice **a,** untreated, **b,** treated with APX2009, **c,** treated with APX2009 and N-AC, and **d,** treated with N-AC categorized based on their functional roles in Fig. 2g. Anti-oxidant features are represented in blue, protein synthesis/translation features in green, DNA damage/peroxidation features in red, and kinase signaling features in black. Strong correlations ($|R| > 0.7$) between features are indicated by black edges, and moderate correlations ($0.5 < |R| \leq 0.7$) are represented by gray edges.

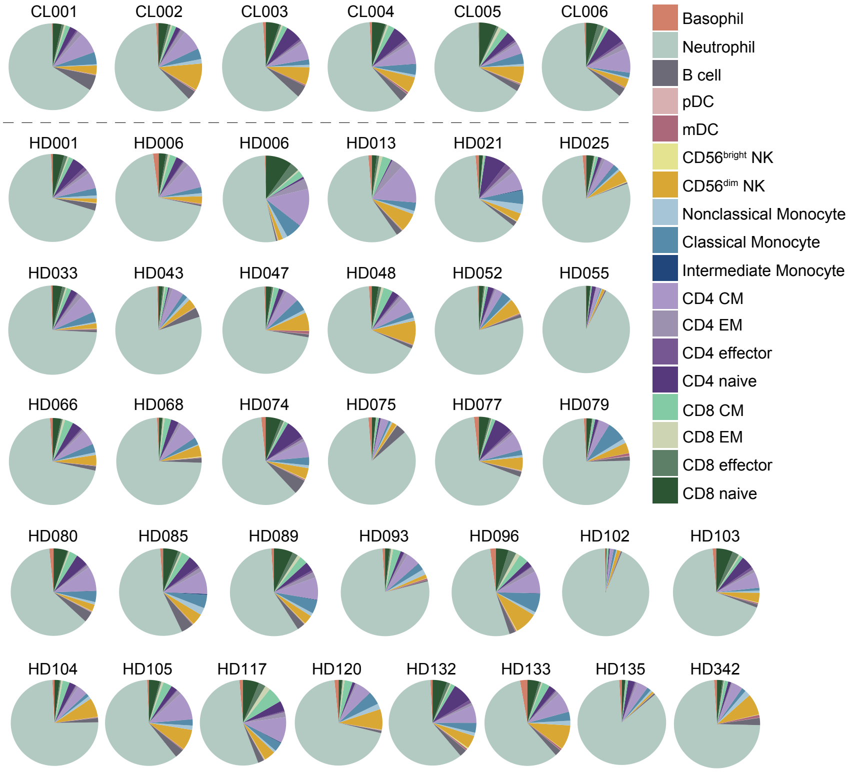


**Supplementary Figure 20| Immune cell subset analysis of whole blood samples from hemodialysis patients.** Pie charts of the proportions of 18 immune cell types in 6 healthy control donors (CL) and 33 hemodialysis patients (HD).

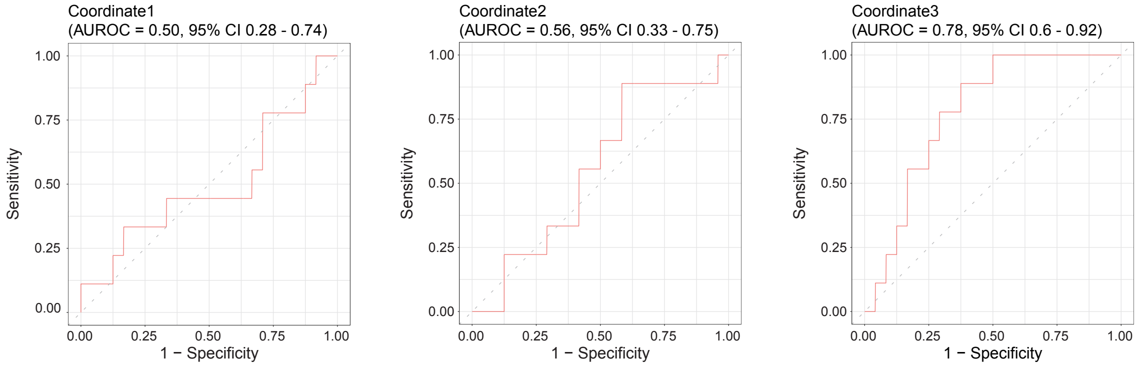


**Supplementary Figure 21| AUROC analysis of the values of different coordinates for prediction of sepsis in hemodialysis patients.** AUROC and associated 95% confidence intervals calculated for performances of multidimensional scaling coordinates 1, 2, and 3 in predicting the risk of sepsis during follow-up.

# Supplementary Table 1 Screening of 103 commercial antibodies for single-cell signaling network under redox stress profiling

| Protein | Clone | catalog | Reactivity | Supplier | Application | Gene | UniProtKB | localization | QC_STDEV |
|---|---|---|---|---|---|---|---|---|---|
| 53bp1 | polyclonal | NB100-304 | Hu, Ms | Novus | WB, IHC, FC | TP53BP1 | Q12888 | Nucleus | pass |
| ACOX1 | EPR19038 | ab184032 | Hu, Ms | abcam | WB, IHC, FC | ACOX1 | Q15067 | Peroxisome | fail |
| ACOX3 | polyclonal | GTX115077 | Hu, Ms | GeneTex | WB, IHC | ACOX3 | O15254 | Peroxisome | pass |
| ALOX12 | polyclonal | E-AB-66327 | Hu, Ms | Elabscience | IHC | ALOX12 | P18054 | Cytoplasm | pass |
| ALOX15 | polyclonal | E-AB-16237 | Hu, Ms | Elabscience | IHC | ALOX15 | P16050 | Cytoplasm | pass |
| ALOX5 | polyclonal | E-AB-67417 | Hu, Ms | Elabscience | IHC | ALOX5 | P09917 | Cytoplasm | pass |
| alpha-Synuclein | 24.8 | NBP1-26380 | Hu, Ms | Novus | IHC | SNCA | P37840 | Cell membrane, Cytoplasm | pass |
| ALR | polyclonal | 11293-1-AP | Hu, Ms | proteintech | WB, IHC, FC | GFER | P56213 | Mitochondria | pass |
| AOC2 | polyclonal | DF14100 | Hu, Ms | Affinity biotech | IHC | AOC2 | O75106 | Cell membrane, Cytoplasm | pass |
| AOC3 | 7-88 | GTX54403 | Ms | GeneTex | IHC | AOC3 | O70423 | Cell membrane | pass |
| AOFA | EPR7101 | ab126751 | Hu, Ms | abcam | WB, IHC, FC | MAOA | P21397 | Mitochondria | fail |
| AOFB | polyclonal | GTX105970 | Hu, Ms | GeneTex | WB, IHC | MAOB | P27338 | Mitochondria | pass |
| AOX1 | polyclonal | E-AB-10765 | Hu, Ms | Elabscience | WB, IHC | AOX1 | Q06278 | Cytoplasm | pass |
| AQP1 | polyclonal | GTX132870 | Hu, Ms | GeneTex | WB, IHC | AQP1 | P29972 | Cell membrane | pass |
| AQP3 | F-1 | sc-518001 | Hu, Ms | Santa cruz | WB, IHC | AQP3 | Q8R2N1 | Cell membrane | fail |
| AQP5 | polyclonal | E-AB-68433 | Hu, Ms | Elabscience | WB, IHC | AQP5 | Q9WTY4 | Cell membrane | fail |
| AQP8 | polyclonal | DF9224 | Hu, Ms | Affinity biotech | WB, IHC | AQP8 | O94778 | Cell membrane | pass |
| AQP9 | polyclonal | tcua6606 | Hu | Taiclone | WB, IHC | AQP9 | O43315 | Cell membrane | pass |
| CaMKII | polyclonal | GTX36254 | Hu, Ms | GeneTex | WB, IHC | CaMKII | Q00168 | Cytoplasm | pass |
| catalase | H-9 | sc-271803 | Hu, Ms | Santa cruz | WB, IHC | CAT | P04040 | Peroxisome | pass |
| CCS | H-7 | sc-55561 | Hu, Ms | Santa cruz | WB, IHC | CCS | O14618 | Cytoplasm | pass |
| CD163 | EPR19518 | ab213612 | Hu, Ms | abcam | WB, IHC, FC | Cd163 | Q2VLH6 | Cell membrane | pass |
| CD204 | 2F8 | MCA1322 | Ms | BIO RAD | WB, IHC, FC | MSR1 | P21757 | Cell membrane | fail |
| CD36 | polyclonal | 18836-1-AP | Hu | proteintech | WB, IHC, FC | Cd36 | Q08857 | Cell membrane | pass |
| COX1 | polyclonal | GTX113259 | Hu, Ms | GeneTex | WB, IHC | MT-CO1 | P00395 | Cell membrane | fail |
| COX17 | A-8 | sc-393617 | Hu | Santa cruz | WB, IHC | COX17 | Q14061 | Mitochondria | fail |
| COX2(Arigo) | polyclonal | ARG56491 | Hu, Ms | Arigo | WB, IHC | PTGS2 | P35354 | Cell membrane | fail |
| COX2(GeneTex) | polyclonal | GTX100656 | Hu, Ms | GeneTex | WB, IHC | PTGS2 | P35354 | Cell membrane | fail |
| CP2D6 | polyclonal | 17868-1-AP | Hu, Ms | proteintech | WB, IHC | CYP2D6 | P10635 | Cell membrane | pass |
| CP2E1 | polyclonal | E-AB-14909 | Hu, Ms | Elabscience | WB, IHC | CYP2E1 | P05181 | Cell membrane | pass |
| CP3A4 | polyclonal | 18227-1-AP | Hu, Ms | proteintech | WB, IHC | CYP3A4 | P08684 | Cell membrane | pass |
| CP4AB | EPR8276 | ab140635 | Hu, Ms | abcam | WB, IHC | CYP4A11 | Q02928 | Cell membrane | pass |
| CRABP1 | C-1 | MA3-813 | Hu, Ms | Invitrogen | WB, IHC, FC | Crabp1 | P62965 | Cell membrane | fail |
| DUOX2 | E-8 | sc-398681 | Hu, Ms | Santa cruz | WB, IHC | DUOX2 | Q9NRD8 | Cell membrane | pass |
| DUSP1 | polyclonal | AF5286 | Hu, Ms | Affinity biotech | WB, IHC | DUSP1 | P28562 | Nucleus | pass |
| EPX | polyclonal | tcua11824 | Hu | Taiclone | WB | EPX | P11678 | Cytoplasm | pass |
| ERO1A | polyclonal | GTX112589 | Hu, Ms | GeneTex | WB, IHC | ERO1A | Q96HE7 | ER | fail |
| ERO1B | polyclonal | 11261-2-AP | Hu, Ms | proteintech | WB, IHC | ERO1B | Q86YB8 | ER | pass |
| FOXO1 | polyclonal | 18592-1-AP | Hu, Ms | proteintech | WB, IHC, FC | FOXO1 | Q12778 | Nucleus | fail |
| FOXO3 | D-12 | sc-48348 | Hu, Ms | Santa cruz | WB, IHC | FOXO3 | Q9WVH4 | Nucleus | fail |
| FOXO4 | polyclonal | 21535-1-AP | Hu, Ms | proteintech | WB, IHC | FOXO4 | Q9WVH3 | Nucleus | pass |
| GAB1 | polyclonal | E-AB-66965 | Hu | Elabscience | IHC | GAB1 | Q13480 | Cytoplasm | pass |
| GPX1/2 | B-6 | sc-133160 | Hu, Ms | Santa cruz | WB, IHC | GPX1 | P07203 | Cytoplasm | fail |
| GPX4 | E-12 | sc-166570 | Hu, Ms | Santa cruz | WB, IHC | GPX4 | P36969 | Mitochondria | pass |
| GR | C-10 | sc-133245 | Hu, Ms | Santa cruz | WB, IHC | GSR | P00390 | Cytoplasm | pass |
| GSS | EPR6563 | ab133592 | Hu, Ms | abcam | WB, IHC, FC | GSS | P48637 | Cytoplasm | fail |
| HAOX1 (Elab) | 3B2 | E-AB-22108 | Hu, Ms | proteintech | WB, IHC | HAO1 | Q9UJM8 | Peroxisome | pass |
| HAOX1 (proteintech) | polyclonal | 25056-1-AP | Hu, Ms | proteintech | WB, IHC | HAO1 | Q9UJM8 | Peroxisome | fail |
| HAOX2 | 3D3 | ab104836 | Hu | abcam | WB, IHC, FC | HAO2 | Q9NYQ2 | Peroxisome | pass |
| HO-1 | polyclonal | ADI-SPA-895-D | Hu, Ms | Enzo | WB, IHC, FC | Hmox1 | P06762 | ER | pass |
| HSP70 | 3A3 | sc-32239 | Hu, Ms | Santa cruz | WB, IHC | HSPBP1 | Q9NZL4 | Cytoplasm | pass |
| KEAP1 | polyclonal | 10503-2-AP | Hu, Ms | proteintech | WB, IHC | KEAP1 | Q9Z2X8 | Cytoplasm | pass |
| LOX | EPR4025 | ab174316 | Hu, Ms | abcam | WB, IHC, FC | LOX | P28300 | Extracellular space | pass |
| LPO | polyclonal | tcua7164 | Ms | Taiclone | WB, IHC | LPO | P22079 | Fluids | pass |
| MDM2 | SMP14 | sc-965 | Hu, Ms | Santa cruz | WB, IHC | MDM2 | P23804 | Nucleus | pass |
| MTH1 | polyclonal | NB100-109 | Hu, Ms | Novus | WB, IHC | NUDT1 | P36639 | Cytoplasm | pass |
| NAMPT | polyclonal | 11776-1-AP | Hu, Ms | proteintech | WB, IHC | NAMPT | P43490 | Cytoplasm | pass |
| NMNAT-2 | B-10 | sc-515206 | Hu, Ms | Santa cruz | WB, IHC | NMNAT2 | Q9BZQ4 | Cytoplasm | pass |
| NNT | polyclonal | 13442-2-AP | Hu, Ms | proteintech | WB, IHC | NNT | Q13423 | Mitochondria | pass |
| NOS1 | A-11 | sc-5302 | Hu, Ms | Santa cruz | WB, IHC | NOS1 | P29475 | Cytoplasm | pass |
| NOS3 (eNOS) | A-9 | sc-376751 | Hu, Ms | Santa cruz | WB, IHC | NOS3 | P29474 | Cell membrane, Golgi | pass |
| NOX1 (p47phox) | D-10 | sc-17845 | Hu, Ms | Santa cruz | WB, IHC | NOX1 | Q9Y5S8 | Cell membrane | pass |
| NOX2 (gp91phox) | 54.1 | sc-130543 | Hu, Ms | Santa cruz | WB, IHC | CYBB | P04839 | Cell membrane | fail |
| NRF2 | polyclonal | 16396-1-AP | Hu, Ms | proteintech | WB, IHC, FC | NFE2L2 | Q16236 | Nucleus | pass |
| Nurr1 | polyclonal | DF12678 | Hu | Affinity biotech | WB | NR4A2 | P43354 | Nucleus | pass |
| OGG1 | polyclonal | NB100-106 | Hu, Ms | Novus | WB, IHC, FC | OGG1 | O15527 | Nucleus | fail |
| OLR1 | polyclonal | 11837-1-AP | Hu, Ms | proteintech | WB, IHC, FC | OLR1 | P78380 | Cell membrane | pass |
| OXDA | polyclonal | 13273-1-AP | Hu, Ms | proteintech | WB, IHC | DAO | P14920 | Peroxisome | pass |
| OXDD | polyclonal | 13682-1-AP | Hu, Ms | proteintech | WB | DDO | Q99489 | Peroxisome | pass |
| OXLA | polyclonal | L4669 | Hu | Sigma-Aldrich | WB | LOX | P28300 | Extracellular space | pass |
| p22-phox | 44.1 | sc-130550 | Hu, Ms | Santa cruz | WB, IHC | CYBA | P13498 | Cell membrane | fail |
| p53 | DO-1 | sc-126 | Hu, Ms | Santa cruz | WB, IHC | TP53 | P04637 | Nucleus | pass |
| PAO | C-3 | sc-166185 | Hu, Ms | Santa cruz | WB, IHC | PAOX | Q6QHF9 | Peroxisome | fail |
| PAX8 | PAX8R1 | sc-81353 | Hu, Ms | Santa cruz | WB, IHC | PAX8 | Q06710 | Nucleus | fail |
| PCYOX1 | polyclonal | 18868-1-AP | Hu, Ms | proteintech | IHC | PCYOX1 | Q9UHG3 | ER | fail |
| PCYOXL | polyclonal | NBP1-81121 | Hu | Novus | WB, IHC | PCYOX1L | Q8NBM8 | ER | pass |
| PDI | RL90 | MA3-019 | Hu, Ms | Invitrogen | WB, IHC, FC | P4HB | P07237 | ER | pass |
| PHD2 | H-8 | sc-271835 | Hu, Ms | Santa cruz | WB, IHC | Egln3 | Q91UZ4 | Nucleus | fail |
| PNPO | polyclonal | E-AB-19759 | Hu, Ms | Elabscience | WB, IHC | PNPO | Q9NVS9 | Mitochondria | pass |
| PRDX1 | 2J5 | E-AB-22067 | Hu, Ms | Elabscience | WB, IHC | PRDX1 | Q06830 | Cytoplasm | pass |
| PRDX2(Elab) | polyclonal | E-AB-15831 | Hu, Ms | Elabscience | WB, IHC | PRDX2 | P32119 | Cytoplasm | pass |
| PRDX2(santa cruz) | A-2 | sc-515428 | Hu, Ms | Santa cruz | WB, IHC | PRDX2 | P32119 | Cytoplasm | fail |
| PRDX4 | polyclonal | E-AB-19430 | Hu, Ms | Elabscience | WB, IHC | PRDX4 | Q13162 | ER | pass |
| PRDX5 | polyclonal | E-AB-10557 | Hu, Ms | Elabscience | IHC | PRDX5 | P30044 | Mitochondria, Peroxisomes | fail |
| PRX-SO3 | polyclonal | ab16830 | Hu | abcam | WB, IHC | PRDX1, 2, 3, 4 | P30048 | Cytoplasm | pass |
| PXDN | polyclonal | tcua352 | Hu | Taiclone | WB, IHC | pxn-1 | Q1ENI8 | Extracellular space | pass |
| QSOX1 | polyclonal | 12713-1-AP | Hu, Ms | proteintech | WB, IHC | QSOX1 | O00391 | Golgi | pass |
| RBCK1 | polyclonal | GTX115451 | Hu, Ms | GeneTex | IHC | RBCK1 | Q9BYM8 | Cytoplasm | fail |
| Ref-1 | C-4 | sc-17774 | Hu, Ms | Santa cruz | WB, IHC | APEX1 | P27695 | Nucleus | pass |
| SEPP1 | B-9 | sc-376858 | Hu, Ms | Santa cruz | WB, IHC | SELENOP | P49908 | Cytoplasm | pass |
| SIRT1 | B-7 | sc-74465 | Hu, Ms | Santa cruz | WB, IHC | SIRT1 | Q96EB6 | Nucleus | fail |
| SIRT3 | F-10 | sc-365175 | Hu, Ms | Santa cruz | WB, IHC | SIRT3 | Q9NTG7 | Mitochondria | fail |
| SOD1 | 24 | sc-101523 | Hu, Ms | Santa cruz | WB, IHC | SOD1 | P00441 | Cytoplasm | fail |
| SOD2K68Ac | EPVANR2 | ab137037 | Hu, Ms | abcam | WB, IHC | SOD2 | P04179 | Mitochondria | pass |
| SOD3 | 4G11G6 | sc-101338 | Hu, Ms | Santa cruz | WB, IHC | SOD3 | P08294 | Extracellular space | fail |
| SOX2 | polyclonal | E-AB-52071 | Hu, Ms | Elabscience | IHC | SOX2 | P48431 | Nucleus | pass |
| SOX3 | polyclonal | E-AB-53348 | Hu, Ms | Elabscience | IHC | SOX3 | P41225 | Nucleus | pass |
| SUOX | polyclonal | GTX105902 | Hu, Ms | GeneTex | WB, IHC | SUOX | P51687 | Mitochondria | pass |
| TRX | polyclonal | 14999-1-AP | Hu, Ms | proteintech | WB, IHC, FC | TXN | P10599 | Cytoplasm, Nucleus | pass |
| TRXR1 | B-2 | sc-28321 | Hu, Ms | Santa cruz | WB, IHC | Txnrd1 | Q9JMH6 | Cytoplasm | pass |
| UCP1 | polyclonal | ARG55180 | Hu, Ms | Arigo | WB, IHC | UCP1 | P04633 | Mitochondria | pass |
| UCP2 | G-6 | sc-390189 | Hu, Ms | Santa cruz | WB, IHC | PHB2 | Q99623 | Mitochondria | pass |
| XO | A-3 | sc-398548 | Hu, Ms | Santa cruz | WB, IHC | XDH | P47989 | Cytoplasm | fail |

Supplementary Table 2 List of signaling and phenotypic antibodies used across different SN-ROP panels

| Target | Metal | Element | Clone | Supplier | Panel | Reactivity |
|---|---|---|---|---|---|---|
| mTOR | 112 | 112 | D8C1 | Cell signaling | all | Hu, Ms |
| pS6 | 113 | 113 | D57.2.2E | Fluidigm | all | Hu, Ms |
| HIF1α | 114 | 114 | EP1215Y | R&D | all | Hu, Ms |
| pNFκB | 166 | 166 | E379 | Fluidigm | all | Hu, Ms |
| pAkt | 152 | 152 | D9E | Fluidigm | all | Hu, Ms |
| pERK | 171 | 171 | 20A | Fluidigm | all | Hu, Ms |
| p38MAPK | 156 | 156 | 36/p38 | Fluidigm | all | Hu, Ms |
| c-Jun | 175 | 175 | D5H2 | Cell signaling | all | Hu, Ms |
| CD4 | 174 | Yb | SK3 | Biolegend | Whole blood CyTOF panel | Hu |
| CD3 | 170 | Er | UCHT1 | Biolegend | Whole blood CyTOF panel | Hu |
| CD57 | 139 | La | HCD57 | Biolegend | Whole blood CyTOF panel | Hu |
| CD36 | 152 | Sm | 5-271 | Fluidigm | Whole blood CyTOF panel | Hu |
| CD38 | 163 | Dy | HIT2 | Biolegend | Whole blood CyTOF panel | Hu |
| CD45RA | 153 | Eu | HI100 | Fluidigm | Whole blood CyTOF panel | Hu |
| CD123 | 151 | Eu | 6H6 | Biolegend | Whole blood CyTOF panel | Hu |
| CD14 | 159 | Tb | M5E2 | Biolegend | Whole blood CyTOF panel | Hu |
| CD11c | 166 | Er | Bu15 | Invitrogen | Whole blood CyTOF panel | Hu |
| CD235 | 173 | Yb | HIR2 | Biolegend | Whole blood CyTOF panel | Hu |
| CD19 | 142 | Nd | HIB19 | Biolegend | Whole blood CyTOF panel | Hu |
| p53 | 171 | Yb | DO-1 | Santa Cruz | Whole blood CyTOF panel | Hu |
| CD8 | 176 | Yb | OKT8 | Thermo | Whole blood CyTOF panel | Hu |
| CD45 | 89 | Y | HI30 | Fluidigm | Whole blood CyTOF panel | Hu |
| HLA-DR | 175 | Lu | L243 | Biolegend | Whole blood CyTOF panel | Hu |
| CD16 | 209 | Bi | 3G8 | Biolegend | Whole blood CyTOF panel | Hu |
| CD7 | 154 | Sm | 6B7 | Biolegend | Whole blood CyTOF panel | Hu |
| CCR7 | 155 | Gd | G043H7 | Biolegend | Whole blood CyTOF panel | Hu |
| CD56 | 162 | Dy | NCAM16.2 | BD | Whole blood CyTOF panel | Hu |
| CD66b | 140 | Ce | G10F5 | Biolegend | Whole blood CyTOF panel | Hu |
| CD137 | 170 | Er | 17B5 | Biolegend | OT-1 | Ms |
| LAG3 | 174 | Yb | C9B7W | Biolegend | OT-1 | Hu, Ms |
| TOX | 154 | Sm | TXRX10 | ebioscience | OT-1 | Hu, Ms |
| CTLA4 | 173 | Yb | UC10-4B9 | Biolegend | OT-1 | Ms |
| TCF1/7 | 142 | Nd | C63D9 | Cell signaling | OT-1 | Hu, Ms |
| EOMES | 161 | Dy | Dan11mag | eBioscience | OT-1 | Hu, Ms |
| CD62L | 163 | Dy | NCAM16.2 | Biolegend | OT-1 | Ms |
| CD8 | 155 | Gd | 53-6.7 | Biolegend | OT-1 | Ms |
| PD1 | 159 | Tb | 29F.1A12 | Biolegend | OT-1 | Hu, Ms |
| TIM3 | 162 | Dy | polyclonal | Biolegend | OT-1 | Hu, Ms |
| CD3 | 140 | Ce | 17A2 | Biolegend | OT-1 | Ms |
| CD90 | 163 | Dy | G7 | BD | MC38 | Ms |
| Ly6G | 139 | La | 1A8 | Biolegend | MC38 | Ms |
| T-bet | 161 | Dy | O4-46 | BD | MC38 | Ms |
| CD19 | 173 | Yb | 6D5 | Fluidigm | MC38 | Ms |
| CD11b | 140 | Ce | M1/70 | Biolegend | MC38 | Ms |
| CD3 | 152 | Sm | 145-2C11 | Fluidigm | MC38 | Ms |
| Biotin (CAR-T) | 165 | Ho | C-10 | Biolegend | CAR-T | Hu, Ms |
| CTLA4 | 170 | Er | polyclonal | Fluidigm | CAR-T | Hu |
| PD1 | 175 | Lu | EH12.2H7 | Biolegend | CAR-T | Hu |
| CD19 | 163 | Dy | HIB19 | Biolegend | CAR-T | Hu |
| EOMES | 173 | Yb | WD1928 | Invitrogen | CAR-T | Hu |
| LAG3 | 150 | Nd | 11C3C65 | Biolegend | CAR-T | Hu |
| CD3 | 113 | In | UCHT1 | Biolegend | CAR-T | Hu |
| CD278 | 151 | Eu | DX29 | DVC | CAR-T | Hu, Ms |
| TIM3 | 154 | Sm | 344823 | R&D system | CAR-T | Hu |
| HLA-DR | 140 | Ce | L243 | Biolegend | CAR-T | Hu |
| Biotin (Pimonidazole) | 165 | Ho | Hypoxyprobe, Inc. | Biolegend | Hypoxia | Hu, Ms |
| CD4 | 141 | Pr | EPR6855 | Abcam | HCC | Hu |
| GR | 155 | Gd | C-10 | Santa Cruz | HCC | Hu |
| CD56 | 163 | Dy | NCAM16.2 | BD | HCC | Hu |
| CD14 | 209 | Bi | M5E2 | Biolegend | HCC | Hu |
| EOMES | 154 | Sm | WD1928 | Invitrogen | HCC | Hu |
| CD68 | 112 | Cd | Y1/82A | Biolegend | HCC | Hu |
| CD11c | 159 | Tb | Bu15 | Invitrogen | HCC | Hu |
| PD1 | 174 | Yb | EH12.2H7 | Fluidigm | HCC | Hu |
| TCF1/7 | 151 | Eu | C63D9 | Cell signaling | HCC | Hu, Ms |
| CTLA4 | 162 | Dy | polyclonal | Thermo | HCC | Hu |
| CD8 | 139 | La | RTA-T8 | Biolegend | HCC | Hu |
| CD16 | 165 | Ho | 3G8 | Biolegend | HCC | Hu |

# Supplementary Table 3 Jurkat cells CyTOF panel

| Protein | Metal | Element | Clone | Concentration [ug/ml] |
|---|---|---|---|---|
| BC1 | 102 | Pd | | |
| BC2 | 104 | Pd | | |
| BC3 | 105 | Pd | | |
| BC4 | 106 | Pd | | |
| BC5 | 108 | Pd | | |
| BC6 | 110 | Pd | | |
| NNT | 111 | Cd | polyclonal | 2 |
| mTOR | 112 | Cd | 7C10 | 0.5 |
| pS6 | 113 | In | N7-548 | 0.5 |
| KEAP1 | 114 | Cd | polyclonal | 1 |
| HSP70 | 115 | In | W27 | 1 |
| PCYXL | 116 | Cd | polyclonal | 2 |
| PRDX4 | 141 | Pr | polyclonal | 0.25 |
| SOD2(K68Ac) | 143 | Nd | EPVANR2 | 4 |
| 53bp1 | 144 | Nd | polyclonal | 1 |
| GPX4 | 145 | Nd | E-12 | 2 |
| NRF2 | 146 | Nd | polyclonal | 1 |
| HIF1α | 147 | Sm | 241812 | 1 |
| PDI | 148 | Nd | RL90 | 1 |
| HO1 | 149 | Sm | polyclonal | 1 |
| MTH1 | 150 | Nd | polyclonal | 1 |
| OLR1 | 151 | Eu | polyclonal | 1 |
| pAkt | 152 | Sm | D9E | 1 |
| CD36 | 153 | Eu | polyclonal | 1 |
| p38MAPK | 156 | Gd | D3F9 | 1 |
| Catalase | 157 | Gd | H-9 | 1 |
| ACOX3 | 158 | Gd | polyclonal | 1 |
| AQP8 | 160 | Gd | polyclonal | 1 |
| Ref/APE1 | 164 | Dy | C-4 | 2 |
| GR | 165 | Ho | C-10 | 1 |
| pNFκB | 166 | Er | K10-895.12.50 | 1 |
| ERO1B | 167 | Er | polyclonal | 4 |
| QSOX1 | 168 | Er | polyclonal | 1 |
| oxPTP | 169 | Tm | 335636 | 1 |
| pERK | 171 | Yb | D13.14.4E | 1 |
| oxDJ1 | 172 | Yb | M149 | 4 |
| c-Jun | 175 | Lu | polyclonal | 2 |
| p53 | 176 | Yb | DO-1 | 1 |
| DNA | 191/193 | Ir | | |
| CD163 | 209 | Bi | C9B7W | 4 |

Supplementary Table 4 Evaluation of cell lineage-specific SN-ROP using whole blood from healthy donors

| ID | Tissue | Age | Sex | Diagnosis |
|---|---|---|---|---|
| 1 | Whole Blood | 46 | male | Healthy |
| 2 | Whole Blood | 48 | male | Healthy |
| 3 | Whole Blood | 36 | male | Healthy |
| 4 | Whole Blood | 34 | female | Healthy |
| 5 | Whole Blood | 26 | male | Healthy |
| 6 | Whole Blood | 27 | female | Healthy |
| 7 | Whole Blood | 27 | male | Healthy |
| 8 | Whole Blood | 25 | female | Healthy |
| 9 | Whole Blood | 27 | female | Healthy |
| 10 | Whole Blood | 29 | female | Healthy |

Supplementary Table 5 Whole blood healthy donors, hemodialysis patients, and healthy control donors CyTOF panel

| Protein | Metal | Element | Clone | Concentration [ug/ml] |
|---|---|---|---|---|
| CD45 | 89 | Y | HI30 | 1 |
| BC1 | 102 | Pd | | |
| BC2 | 104 | Pd | | |
| BC3 | 105 | Pd | | |
| BC4 | 106 | Pd | | |
| BC5 | 108 | Pd | | |
| BC6 | 110 | Pd | | |
| NNT | 111 | Cd | polyclonal | 2 |
| mTOR | 112 | Cd | 7C10 | 0.5 |
| pS6 | 113 | In | N7-548 | 0.5 |
| KEAP1 | 114 | Cd | polyclonal | 1 |
| HSP70 | 115 | In | W27 | 1 |
| PCYXL | 116 | Cd | polyclonal | 2 |
| CD57 | 139 | La | HCD57 | 1:40, then 2 µL |
| CD66b | 140 | Ce | G10F5 | 0.5 |
| PRDX4 | 141 | Pr | polyclonal | 0.25 |
| CD19 | 142 | Nd | HIB19 | 1 |
| SOD2(K68Ac) | 143 | Nd | EPVANR2 | 4 |
| 53bp1 | 144 | Nd | polyclonal | 1 |
| GPX4 | 145 | Nd | E-12 | 2 |
| NRF2 | 146 | Nd | polyclonal | 1 |
| HIF1α | 147 | Sm | 241812 | 1 |
| PDI | 148 | Nd | RL90 | 1 |
| HO1 | 149 | Sm | polyclonal | 1 |
| MTH1 | 150 | Nd | polyclonal | 1 |
| CD123 | 151 | Eu | 6H6 | 1 |
| CD36 | 152 | Sm | 5-271 | 1:10, then 1 µL |
| CD45RA | 153 | Eu | HI100 | 0.5 |
| CD7 | 154 | Sm | 6B7 | 0.25 |
| CCR7 | 155 | Gd | G043H7 | 1 |
| p38MAPK | 156 | Gd | D3F9 | 1 |
| Catalase | 157 | Gd | H-9 | 1 |
| ACOX3 | 158 | Gd | polyclonal | 1 |
| CD14 | 159 | Tb | M5E2 | 0.25 |
| AQP8 | 160 | Gd | polyclonal | 1 |
| CD56 | 162 | Dy | NCAM16.2 | 1 |
| CD38 | 163 | Dy | HIT2 | 1 |
| Ref/APE1 | 164 | Dy | C-4 | 2 |
| GR | 165 | Ho | C-10 | 1 |
| CD11c | 166 | Er | Bu15 | 1 |
| ERO1B | 167 | Er | polyclonal | 4 |
| QSOX1 | 168 | Er | polyclonal | 1 |
| oxPTP | 169 | Tm | 335636 | 1 |
| CD3 | 170 | Er | UCHT1 | 1:10, then 1 µL |
| p53 | 171 | Yb | DO-1 | 1:10, then 0.25 µL |
| oxDJ1 | 172 | Yb | M149 | 4 |
| CD235 | 173 | Yb | HIR2 | 2 |
| CD4 | 174 | Yb | SK3 | 1 |
| HLA-DR | 175 | Lu | L243 | 0.5 |
| CD8 | 176 | Yb | OKT8 | 1.5 |
| DNA | 191/193 | Ir | | |
| CD16 | 209 | Bi | 3G8 | 0.5 |

# Supplementary Table 6 OT-I CD8$^+$ T cell and N-AC CyTOF panel

| Protein | Metal | Element | Clone | Concentration [ug/ml] |
|---|---|---|---|---|
| CD45 | 89 | Y | HI30 | 0.5 |
| BC1 | 102 | Pd | | |
| BC2 | 104 | Pd | | |
| BC3 | 105 | Pd | | |
| BC4 | 106 | Pd | | |
| BC5 | 108 | Pd | | |
| BC6 | 110 | Pd | | |
| NNT | 111 | Cd | polyclonal | 2 |
| mTOR | 112 | Cd | 7C10 | 0.5 |
| pS6 | 113 | In | N7-548 | 0.5 |
| KEAP1 | 114 | Cd | polyclonal | 1 |
| HSP70 | 115 | In | W27 | 1 |
| PCYXL | 116 | Cd | polyclonal | 2 |
| CD3 | 140 | Ce | 17A2 | 1 |
| PRDX4 | 141 | Pr | polyclonal | 0.25 |
| TCF1/7 | 142 | Nd | C63D9 | 1.5 |
| SOD2(K68Ac) | 143 | Nd | EPVANR2 | 2 |
| 53bp1 | 144 | Nd | polyclonal | 1 |
| GPX4 | 145 | Nd | E-12 | 2 |
| NRF2 | 146 | Nd | polyclonal | 1 |
| HIF1α | 147 | Sm | 241812 | 1 |
| PDI | 148 | Nd | RL90 | 1 |
| HO1 | 149 | Sm | polyclonal | 1 |
| MTH1 | 150 | Nd | polyclonal | 1 |
| OLR1 | 151 | Eu | polyclonal | 1 |
| pAkt | 152 | Sm | D9E | 1 |
| CD36 | 153 | Eu | polyclonal | 1 |
| TOX | 154 | Sm | TXRX10 | 1 |
| CD8 | 155 | Gd | 53-6.7 | 1:10, then 1.5 µL |
| p38MAPK | 156 | Gd | D3F9 | 1 |
| Catalase | 157 | Gd | H-9 | 1 |
| ACOX3 | 158 | Gd | polyclonal | 0.5 |
| PD1 | 159 | Tb | 29F.1A12 | 0.5 |
| AQP8 | 160 | Gd | polyclonal | 1 |
| EOMES | 161 | Dy | Dan11mag | 1 |
| TIM3 | 162 | Dy | polyclonal | 0.5 |
| CD62L | 163 | Dy | NCAM16.2 | 0.5 |
| Ref/APE1 | 164 | Dy | C-4 | 2 |
| GR | 165 | Ho | C-10 | 1 |
| pNFκB | 166 | Er | K10-895.12.50 | 1 |
| ERO1B | 167 | Er | polyclonal | 4 |
| QSOX1 | 168 | Er | polyclonal | 1 |
| oxPTP | 169 | Tm | 335636 | 1 |
| CD137 | 170 | Er | 17B5 | 1 |
| pERK | 171 | Yb | D13.14.4E | 1 |
| oxDJ1 | 172 | Yb | M149 | 1 |
| CTLA4 | 173 | Yb | UC10-4B9 | 1 |
| LAG3 | 174 | Yb | C9B7W | 1 |
| c-Jun | 175 | Lu | polyclonal | 2 |
| p53 | 176 | Yb | DO-1 | 1 |
| DNA | 191/193 | Ir | | |
| CD163 | 209 | Bi | C9B7W | 4 |

# Supplementary Table 7 MC38 CyTOF panel

| Protein | Metal | Element | Clone | Concentration [ug/ml] |
|---|---|---|---|---|
| CD45 | 89 | Y | HI30 | 0.5 |
| BC1 | 102 | Pd | | |
| BC2 | 104 | Pd | | |
| BC3 | 105 | Pd | | |
| BC4 | 106 | Pd | | |
| BC5 | 108 | Pd | | |
| BC6 | 110 | Pd | | |
| NNT | 111 | Cd | polyclonal | 2 |
| mTOR | 112 | Cd | 7C10 | 0.5 |
| pS6 | 113 | In | N7-548 | 0.5 |
| KEAP1 | 114 | Cd | polyclonal | 1 |
| HSP70 | 115 | In | W27 | 1 |
| PCYXL | 116 | Cd | polyclonal | 2 |
| Ly6G | 139 | La | 1A8 | 0.5 |
| CD11b | 140 | Ce | M1/70 | 1:10, then 1 µL |
| PRDX4 | 141 | Pr | polyclonal | 0.25 |
| TCF1/7 | 142 | Nd | C63D9 | 1.5 |
| SOD2(K68Ac) | 143 | Nd | EPVANR2 | 4 |
| 53bp1 | 144 | Nd | polyclonal | 1 |
| GPX4 | 145 | Nd | E-12 | 2 |
| NRF2 | 146 | Nd | polyclonal | 1 |
| HIF1α | 147 | Sm | 241812 | 1 |
| PDI | 148 | Nd | RL90 | 1 |
| HO1 | 149 | Sm | polyclonal | 1 |
| MTH1 | 150 | Nd | polyclonal | 1 |
| OLR1 | 151 | Eu | polyclonal | 1 |
| CD3 | 152 | Sm | 145-2C11 | 1:10, then 1 µL |
| CD36 | 153 | Eu | polyclonal | 1 |
| TOX | 154 | Sm | TXRX10 | 1 |
| CD8 | 155 | Gd | 53-6.7 | 1:10, then 1.5 µL |
| p38MAPK | 156 | Gd | D3F9 | 1 |
| Catalase | 157 | Gd | H-9 | 1 |
| ACOX3 | 158 | Gd | polyclonal | 1 |
| PD1 | 159 | Tb | 29F.1A12 | 0.5 |
| AQP8 | 160 | Gd | polyclonal | 1 |
| T-bet | 161 | Dy | O4-46 | 2 |
| TIM3 | 162 | Dy | polyclonal | 0.5 |
| CD90 | 163 | Dy | G7 | 0.5 |
| Ref/APE1 | 164 | Dy | C-4 | 2 |
| NK1.1 | 165 | Ho | PK136 | 1 |
| pNFκB | 166 | Er | K10-895.12.50 | 1 |
| ERO1B | 167 | Er | polyclonal | 4 |
| QSOX1 | 168 | Er | polyclonal | 1 |
| oxPTP | 169 | Tm | 335636 | 1 |
| CD137 | 170 | Er | 17B5 | 1 |
| pERK | 171 | Yb | D13.14.4E | 1 |
| oxDJ1 | 172 | Yb | M149 | 4 |
| CD19 | 173 | Yb | 6D5 | 0.5 |
| LAG3 | 174 | Yb | C9B7W | 1 |
| c-Jun | 175 | Lu | polyclonal | 2 |
| p53 | 176 | Yb | DO-1 | 1 |
| DNA | 191/193 | Ir | | |
| CD163 | 209 | Bi | C9B7W | 4 |

Supplementary Table 8 Demographic information on patients treated with CAR-T cells

| ID | Tissue | Age | Sex | Diagnosis | Cytokine Release Syndrome |
|---|---|---|---|---|---|
| CART1906 | PBMC | 62 | male | CLL | non |
| CART1907 | PBMC | 52 | male | CLL | NA |
| CART1903 | PBMC | 18 | male | ALL | non |
| CART1908 | PBMC | 6 | female | ALL | 3 |
| CART1909 | PBMC | 46 | female | ALL | 1 |
| CART1904 | PBMC | 52 | female | CLL | 2 |
| CART1905 | PBMC | 6 | female | ALL | NA |

# Supplementary Table 9 CAR-T CyTOF panel

| Protein | Metal | Element | Clone | Concentration [ug/ml] |
|---|---|---|---|---|
| CD45 | 89 | Y | HI30 | 1 |
| BC1 | 102 | Pd | | |
| BC2 | 104 | Pd | | |
| BC3 | 105 | Pd | | |
| BC4 | 106 | Pd | | |
| BC5 | 108 | Pd | | |
| BC6 | 110 | Pd | | |
| NNT | 111 | Cd | polyclonal | 2 |
| mTOR | 112 | Cd | 7C10 | 0.5 |
| CD3 | 113 | In | UCHT1 | 1 |
| KEAP1 | 114 | Cd | polyclonal | 1 |
| HSP70 | 115 | In | W27 | 1 |
| PCYXL | 116 | Cd | polyclonal | 2 |
| CD57 | 139 | La | HCD57 | 1:10, then 2 μL |
| HLA-DR | 140 | Ce | L243 | 2 |
| PRDX4 | 141 | Pr | polyclonal | 0.25 |
| TCF1/7 | 142 | Nd | C63D9 | 1.5 |
| SOD2(K68Ac) | 143 | Nd | EPVANR2 | 2 |
| 53bp1 | 144 | Nd | polyclonal | 1 |
| GPX4 | 145 | Nd | E-12 | 2 |
| NRF2 | 146 | Nd | polyclonal | 1 |
| HIF1α | 147 | Sm | 241812 | 1 |
| PDI | 148 | Nd | RL90 | 1 |
| HO1 | 149 | Sm | polyclonal | 1 |
| LAG3 | 150 | Nd | 11C3C65 | 4 |
| CD278 | 151 | Eu | DX29 | 1:10, then 1 μL |
| CD36 | 152 | Sm | 5-271 | 1:10, then 1 μL |
| CD45RA | 153 | Eu | HI100 | 0.5 |
| TIM3 | 154 | Sm | 344823 | 4 |
| CCR7 | 155 | Gd | G043H7 | 1 |
| p38MAPK | 156 | Gd | D3F9 | 1 |
| Catalase | 157 | Gd | H-9 | 1 |
| ACOX3 | 158 | Gd | polyclonal | 0.5 |
| CD14 | 159 | Tb | M5E2 | 0.25 |
| AQP8 | 160 | Gd | polyclonal | 1 |
| T-bet | 161 | Dy | 4B10 | 1 |
| FoxP3 | 162 | Dy | 236A/E7 | 2 |
| CD19 | 163 | Dy | HIB19 | 0.5 |
| Ref/APE1 | 164 | Dy | C-4 | 2 |
| Biotin | 165 | Ho | C-10 | 1 |
| pNFκB | 166 | Er | K10-895.12.50 | 1 |
| ERO1B | 167 | Er | polyclonal | 4 |
| QSOX1 | 168 | Er | polyclonal | 1 |
| oxPTP | 169 | Tm | 335636 | 1 |
| CTLA4 | 170 | Er | polyclonal | 1 |
| p53 | 171 | Yb | DO-1 | 1:10, then 0.25 μL |
| oxDJ1 | 172 | Yb | M149 | 1 |
| EOMES | 173 | Yb | WD1928 | 2 |
| CD4 | 174 | Yb | SK3 | 1 |
| PD1 | 175 | Lu | EH12.2H7 | 0.5 |
| CD8 | 176 | Yb | OKT8 | 1.5 |
| DNA | 191/193 | Ir | | |
| CD163 | 209 | Bi | C9B7W | 4 |

Supplementary Table 10 Hypoxia OT-I CD8$^{+}$ T cell CyTOF panel

| Protein | Metal | Element | Clone | Concentration [ug/ml] |
|---|---|---|---|---|
| CD45 | 89 | Y | HI30 | 0.5 |
| BC1 | 102 | Pd | | |
| BC2 | 104 | Pd | | |
| BC3 | 105 | Pd | | |
| BC4 | 106 | Pd | | |
| BC5 | 108 | Pd | | |
| BC6 | 110 | Pd | | |
| NNT | 111 | Cd | polyclonal | 2 |
| mTOR | 112 | Cd | 7C10 | 0.5 |
| pS6 | 113 | In | N7-548 | 0.5 |
| KEAP1 | 114 | Cd | polyclonal | 1 |
| HSP70 | 115 | In | W27 | 1 |
| PCYXL | 116 | Cd | polyclonal | 2 |
| CD3 | 140 | Ce | 17A2 | 1 |
| PRDX4 | 141 | Pr | polyclonal | 0.25 |
| TCF1/7 | 142 | Nd | C63D9 | 1.5 |
| SOD2(K68Ac) | 143 | Nd | EPVANR2 | 2 |
| 53bp1 | 144 | Nd | polyclonal | 1 |
| GPX4 | 145 | Nd | E-12 | 2 |
| NRF2 | 146 | Nd | polyclonal | 1 |
| HIF1α | 147 | Sm | 241812 | 1 |
| PDI | 148 | Nd | RL90 | 1 |
| HO1 | 149 | Sm | polyclonal | 1 |
| MTH1 | 150 | Nd | polyclonal | 1 |
| OLR1 | 151 | Eu | polyclonal | 1 |
| pAkt | 152 | Sm | D9E | 1 |
| CD36 | 153 | Eu | polyclonal | 1 |
| TOX | 154 | Sm | TXRX10 | 1 |
| CD8 | 155 | Gd | 53-6.7 | 1:10, then 1.5 µL |
| p38MAPK | 156 | Gd | D3F9 | 1 |
| Catalase | 157 | Gd | H-9 | 1 |
| ACOX3 | 158 | Gd | polyclonal | 0.5 |
| PD1 | 159 | Tb | 29F.1A12 | 0.5 |
| AQP8 | 160 | Gd | polyclonal | 1 |
| EOMES | 161 | Dy | Dan11mag | 1 |
| TIM3 | 162 | Dy | polyclonal | 0.5 |
| CD62L | 163 | Dy | NCAM16.2 | 0.5 |
| Ref/APE1 | 164 | Dy | C-4 | 2 |
| Biotin (Pimonidazole) | 165 | Ho | | 1 |
| pNFκB | 166 | Er | K10-895.12.50 | 1 |
| ERO1B | 167 | Er | polyclonal | 4 |
| QSOX1 | 168 | Er | polyclonal | 1 |
| oxPTP | 169 | Tm | 335636 | 1 |
| CD137 | 170 | Er | 17B5 | 1 |
| pERK | 171 | Yb | D13.14.4E | 1 |
| oxDJ1 | 172 | Yb | M149 | 1 |
| CTLA4 | 173 | Yb | UC10-4B9 | 1 |
| LAG3 | 174 | Yb | C9B7W | 1 |
| c-Jun | 175 | Lu | polyclonal | 2 |
| p53 | 176 | Yb | DO-1 | 1 |
| DNA | 191/193 | Ir | | |
| CD163 | 209 | Bi | C9B7W | 4 |

Supplementary Table 11 Demographic information on hepatocellular carcinoma patients

| ID | Tissue | Age | Sex | Diagnosis | classification |
|---|---|---|---|---|---|
| 6802742 | Liver suspension | 65 | male | Hepatic carcinoma | BCLC B/C |
| 6800075 | Liver suspension | 49 | male | Hepatic carcinoma | BCLC B/C |

Supplementary Table 12 Hepatocellular carcinoma CyTOF panel

| Protein | Metal | Element | Clone | Concentration [ug/ml] |
|---|---|---|---|---|
| CD45 | 89 | Y | HI30 | 1 |
| BC1 | 102 | Pd | | |
| BC2 | 104 | Pd | | |
| BC3 | 105 | Pd | | |
| BC4 | 106 | Pd | | |
| BC5 | 108 | Pd | | |
| BC6 | 110 | Pd | | |
| NNT | 111 | Cd | polyclonal | 2 |
| CD68 | 112 | Cd | Y1/82A | 1 |
| pS6 | 113 | In | N7-548 | 0.5 |
| KEAP1 | 114 | Cd | polyclonal | 1 |
| HSP70 | 115 | In | W27 | 1 |
| PCYXL | 116 | Cd | polyclonal | 2 |
| CD8 | 139 | La | RTA-T8 | 1 |
| CD66b | 140 | Ce | G10F5 | 0.5 |
| CD4 | 141 | Pr | EPR6855 | 1 |
| NKG2A | 142 | Nd | Z199 | 1 |
| SOD2(K68Ac) | 143 | Nd | EPVANR2 | 4 |
| 53bp1 | 144 | Nd | polyclonal | 1 |
| GPX4 | 145 | Nd | E-12 | 2 |
| NRF2 | 146 | Nd | polyclonal | 1 |
| HIF1α | 147 | Sm | 241812 | 1 |
| PDI | 148 | Nd | RL90 | 1 |
| HO1 | 149 | Sm | polyclonal | 1 |
| MTH1 | 150 | Nd | polyclonal | 1 |
| TCF1/7 | 151 | Eu | C63D9 | 0.5 |
| pAkt | 152 | Sm | D9E | 1 |
| CD36 | 153 | Eu | polyclonal | 1 |
| EOMES | 154 | Sm | WD1928 | 1 |
| GR | 155 | Gd | C-10 | 1 |
| p38MAPK | 156 | Gd | D3F9 | 1 |
| Catalase | 157 | Gd | H-9 | 1 |
| ACOX3 | 158 | Gd | polyclonal | 1 |
| CD11c | 159 | Tb | Bu15 | 1 |
| AQP8 | 160 | Gd | polyclonal | 1 |
| CTLA4 | 162 | Dy | polyclonal | 1 |
| CD56 | 163 | Dy | NCAM16.2 | 1:10, then 1 µL |
| Ref/APE1 | 164 | Dy | C-4 | 2 |
| CD16 | 165 | Ho | 3G8 | 0.5 |
| pNFκB | 166 | Er | K10-895.12.50 | 1 |
| ERO1B | 167 | Er | polyclonal | 4 |
| QSOX1 | 168 | Er | polyclonal | 1 |
| oxPTP | 169 | Tm | 335636 | 1 |
| CD3 | 170 | Er | UCHT1 | 1:10, then 1 µL |
| pERK | 171 | Yb | D13.14.4E | 1 |
| oxDJ1 | 172 | Yb | M149 | 4 |
| CD19 | 173 | Yb | HIB19 | 1 |
| PD1 | 174 | Yb | EH12.2H7 | 0.5 |
| c-Jun | 175 | Lu | polyclonal | 2 |
| p53 | 176 | Yb | DO-1 | 1 |
| DNA | 191/193 | Ir | | |
| CD14 | 209 | Bi | M5E2 | 1.5 |

Supplementary Table 13 LCMV (lymphocytic choriomeningitis virus) infection in B6 mice CyTOF panel

| Protein | Metal | Element | Clone | Concentration [ug/ml] |
|---|---|---|---|---|
| CD45 | 89 | Y | HI30 | 0.5 |
| BC1 | 102 | Pd | | |
| BC2 | 104 | Pd | | |
| BC3 | 105 | Pd | | |
| BC4 | 106 | Pd | | |
| BC5 | 108 | Pd | | |
| BC6 | 110 | Pd | | |
| NNT | 111 | Cd | polyclonal | 2 |
| mTOR | 112 | Cd | 7C10 | 0.5 |
| pS6 | 113 | In | N7-548 | 0.5 |
| KEAP1 | 114 | Cd | polyclonal | 1 |
| HSP70 | 115 | In | W27 | 1 |
| PCYXL | 116 | Cd | polyclonal | 2 |
| Ly6G | 139 | La | 1A8 | 0.5 |
| CD11b | 140 | Ce | M1/70 | 1:10, then 1 μL |
| PRDX4 | 141 | Pr | polyclonal | 0.25 |
| TCF1/7 | 142 | Nd | C63D9 | 1.5 |
| SOD2(K68Ac) | 143 | Nd | EPVANR2 | 4 |
| 53bp1 | 144 | Nd | polyclonal | 1 |
| GPX4 | 145 | Nd | E-12 | 2 |
| NRF2 | 146 | Nd | polyclonal | 1 |
| HIF1α | 147 | Sm | 241812 | 1 |
| PDI | 148 | Nd | RL90 | 1 |
| HO1 | 149 | Sm | polyclonal | 1 |
| MTH1 | 150 | Nd | polyclonal | 1 |
| OLR1 | 151 | Eu | polyclonal | 1 |
| CD3 | 152 | Sm | 145-2C11 | 1:10, then 1 μL |
| CD36 | 153 | Eu | polyclonal | 1 |
| TOX | 154 | Sm | TXRX10 | 1 |
| CD8 | 155 | Gd | 53-6.7 | 1:10, then 1.5 μL |
| p38MAPK | 156 | Gd | D3F9 | 1 |
| Catalase | 157 | Gd | H-9 | 1 |
| ACOX3 | 158 | Gd | polyclonal | 1 |
| PD1 | 159 | Tb | 29F.1A12 | 0.5 |
| AQP8 | 160 | Gd | polyclonal | 1 |
| T-bet | 161 | Dy | O4-46 | 2 |
| TIM3 | 162 | Dy | polyclonal | 0.5 |
| CD90 | 163 | Dy | G7 | 0.5 |
| Ref/APE1 | 164 | Dy | C-4 | 2 |
| pNFκB | 166 | Er | K10-895.12.50 | 1 |
| ERO1B | 167 | Er | polyclonal | 4 |
| QSOX1 | 168 | Er | polyclonal | 1 |
| oxPTP | 169 | Tm | 335636 | 1 |
| CD137 | 170 | Er | 17B5 | 1 |
| pERK | 171 | Yb | D13.14.4E | 1 |
| oxDJ1 | 172 | Yb | M149 | 4 |
| CD19 | 173 | Yb | 6D5 | 0.5 |
| LAG3 | 174 | Yb | C9B7W | 1 |
| c-Jun | 175 | Lu | polyclonal | 2 |
| p53 | 176 | Yb | DO-1 | 1 |
| DNA | 191/193 | Ir | | |
| CD163 | 209 | Bi | C9B7W | 4 |

Supplementary Table 14 Demographic information of patients on hemodialysis and healthy controls

| ID | Tissue | Sepsis | Age | Sex | Diagnosis | HD cause | HD_month |
|---|---|---|---|---|---|---|---|
| HD001 | Whole Blood | N | 55 | male | Hemodialysis | Hypertension | 309 |
| HD003 | Whole Blood | N | 68 | female | Hemodialysis | Diabetes | 113 |
| HD006 | Whole Blood | Y | 82 | female | Hemodialysis | Diabetes | 142 |
| HD013 | Whole Blood | N | 62 | female | Hemodialysis | Others | 69 |
| HD021 | Whole Blood | N | 61 | female | Hemodialysis | Hypertension | 64 |
| HD025 | Whole Blood | N | 71 | female | Hemodialysis | Glomerulonephritis | 107 |
| HD033 | Whole Blood | Y | 76 | male | Hemodialysis | Glomerulonephritis | 136 |
| HD043 | Whole Blood | N | 62 | male | Hemodialysis | Glomerulonephritis | 322 |
| HD047 | Whole Blood | Y | 72 | male | Hemodialysis | Glomerulonephritis | 221 |
| HD048 | Whole Blood | Y | 57 | female | Hemodialysis | Hypertension | 64 |
| HD052 | Whole Blood | Y | 73 | male | Hemodialysis | Hypertension | 92 |
| HD055 | Whole Blood | N | 52 | male | Hemodialysis | Hypertension | 88 |
| HD066 | Whole Blood | N | 62 | female | Hemodialysis | Diabetes | 243 |
| HD068 | Whole Blood | N | 51 | male | Hemodialysis | Diabetes | 54 |
| HD074 | Whole Blood | Y | 66 | female | Hemodialysis | Diabetes | 65 |
| HD075 | Whole Blood | N | 61 | female | Hemodialysis | Hypertension | 53 |
| HD077 | Whole Blood | N | 59 | male | Hemodialysis | Others | 114 |
| HD079 | Whole Blood | N | 63 | female | Hemodialysis | Hypertension | 57 |
| HD080 | Whole Blood | N | 36 | female | Hemodialysis | Glomerulonephritis | 76 |
| HD085 | Whole Blood | N | 60 | male | Hemodialysis | Others | 139 |
| HD089 | Whole Blood | N | 51 | male | Hemodialysis | Diabetes | 88 |
| HD093 | Whole Blood | N | 59 | female | Hemodialysis | Others | 179 |
| HD096 | Whole Blood | N | 64 | male | Hemodialysis | Glomerulonephritis | 90 |
| HD102 | Whole Blood | Y | 63 | female | Hemodialysis | Others | 86 |
| HD103 | Whole Blood | N | 55 | female | Hemodialysis | Glomerulonephritis | 60 |
| HD104 | Whole Blood | N | 53 | female | Hemodialysis | Others | 75 |
| HD105 | Whole Blood | N | 55 | male | Hemodialysis | Glomerulonephritis | 90 |
| HD117 | Whole Blood | N | 64 | male | Hemodialysis | Others | 267 |
| HD120 | Whole Blood | N | 73 | female | Hemodialysis | Hypertension | 95 |
| HD132 | Whole Blood | Y | 57 | female | Hemodialysis | Others | 145 |
| HD133 | Whole Blood | Y | 56 | male | Hemodialysis | Diabetes | 84 |
| HD135 | Whole Blood | N | 61 | male | Hemodialysis | Others | 202 |
| HD342 | Whole Blood | N | 80 | female | Hemodialysis | Hypertension | 38 |
| CL001 | Whole Blood |  | 49 | male | Healthy |  |  |
| CL002 | Whole Blood |  | 54 | female | Healthy |  |  |
| CL003 | Whole Blood |  | 53 | male | Healthy |  |  |
| CL004 | Whole Blood |  | 39 | female | Healthy |  |  |
| CL005 | Whole Blood |  | 33 | female | Healthy |  |  |
| CL006 | Whole Blood |  | 62 | male | Healthy |  |  |

Supplementary Table 15 Correlation of SN-ROP features with coordinate 3 of the multidimensional scaling for unsupervised clustering

| Immunometabolic features | Spearman correlation coefficient ρ | p value |
|---|---|---|
| T cell related | | |
| CD4CM_GPX4 | -0.6531 | <0.001 |
| CD4CM_NRF2 | -0.5679 | <0.001 |
| CD4CM_PRDX4 | -0.6019 | <0.001 |
| CD4eff_GPX4 | -0.6608 | <0.001 |
| CD4EM_GPX4 | -0.6674 | <0.001 |
| CD4naive_GPX4 | -0.6430 | <0.001 |
| CD8CM_AQP8 | 0.6695 | <0.001 |
| CD8CM_GPX4 | -0.6932 | <0.001 |
| CD8CM_GR | 0.6892 | <0.001 |
| CD8CM_p53 | 0.6649 | <0.001 |
| CD8CM_PRDX4 | -0.5538 | <0.001 |
| CD8CM_Ref_APE | 0.6885 | <0.001 |
| CD8eff_AQP8 | 0.6150 | <0.001 |
| CD8eff_GR | 0.6039 | <0.001 |
| CD8eff_oxPTP | 0.6237 | <0.001 |
| CD8eff_QSOX1 | 0.6494 | <0.001 |
| CD8eff_Ref_APE | 0.5812 | <0.001 |
| CD8EM_GPX4 | -0.6544 | <0.001 |
| CD8EM_GR | 0.6775 | <0.001 |
| CD8EM_Ref_APE | 0.5892 | <0.001 |
| CD8naive_AQP8 | 0.6441 | <0.001 |
| CD8naive_GR | 0.6827 | <0.001 |
| CD8naive_HIF1a | 0.5652 | <0.001 |
| CD8naive_oxPTP | 0.7139 | <0.001 |
| CD8naive_p53 | 0.6437 | <0.001 |
| CD8naive_QSOX1 | 0.6858 | <0.001 |
| CD8naive_Ref_APE | 0.6731 | <0.001 |
| CD8naive_GPX4 | -0.5461 | 0.001 |
| CD4naive_NRF2 | -0.5376 | 0.001 |
| CD8eff_p53 | 0.5367 | 0.001 |
| CD4EM_GR | 0.5358 | 0.001 |
| CD8CM_QSOX1 | 0.5308 | 0.001 |
| CD4CM_GR | 0.5203 | 0.002 |
| CD8CM_oxPTP | 0.5010 | 0.003 |
| CD4CM_Ref_APE | 0.4866 | 0.004 |
| CD4naive_PRDX4 | -0.4865 | 0.004 |
| CD8EM_p53 | 0.4743 | 0.005 |
| CD8eff_GPX4 | -0.4719 | 0.006 |
| Others | | |
| B_p38MAPK | -0.6695 | <0.001 |
| baso_ERO1B | -0.7196 | <0.001 |
| baso_p38MAPK | -0.7443 | <0.001 |
| bright_ERO1B | -0.6320 | <0.001 |
| classical_GPX4 | -0.5722 | <0.001 |
| dim_ERO1B | -0.5588 | <0.001 |
| IFNg | -0.6461 | <0.001 |
| classical_PRDX4 | -0.5307 | 0.001 |
| alter_GPX4 | -0.5264 | 0.002 |
| baso_pS6 | -0.5144 | 0.002 |
| baso_Ref_APE | -0.5110 | 0.002 |
| B_ERO1B | -0.5064 | 0.003 |

Spearman correlation analysis was performed to evaluate associations between SN-ROP marker expression within immune cell subsets and MDS coordinate 3 values. Reported statistics include Spearman's correlation coefficient (*ρ*) and two-sided *P* values. All *P* values were < 0.001, indicating strong and statistically significant correlations. Multiple comparisons were not adjusted due to the exploratory nature of the analysis.

# Supplementary Table 16 Comparison of model performance metrics based on F1 Scores and macro average for multi-class classification

| | Class 0 F1-Score | Class 1 F1-Score | Class 2 F1-Score | Class 3 F1-Score | Class 4 F1-Score | Class 5 F1-Score | Accuracy | Macro Average | Weighted Avg |
|---|---|---|---|---|---|---|---|---|---|
| CatBoost Classifier | 0.9805 | 0.8055 | 0.6882 | 0.6744 | 0.6961 | 0.6008 | 0.7327 | 0.7409 | 0.732 |
| XGBoost | 0.9773 | 0.7854 | 0.6806 | 0.666 | 0.6922 | 0.5935 | 0.7256 | 0.7325 | 0.7248 |
| Random Forest | 0.9666 | 0.7023 | 0.6246 | 0.6227 | 0.6752 | 0.5347 | 0.6862 | 0.6877 | 0.6842 |
| Neural Network | 0.9602 | 0.723 | 0.6299 | 0.5865 | 0.6454 | 0.5322 | 0.6728 | 0.6795 | 0.6704 |
| Quadratic Discriminant Analysis | 0.9409 | 0.6749 | 0.6139 | 0.6218 | 0.6277 | 0.5171 | 0.6668 | 0.6661 | 0.6659 |
| AdaBoost | 0.9585 | 0.6429 | 0.6003 | 0.5496 | 0.6169 | 0.4976 | 0.6418 | 0.6443 | 0.6398 |
| Naive Bayes | 0.9332 | 0.5693 | 0.5522 | 0.5565 | 0.5539 | 0.4901 | 0.6121 | 0.6092 | 0.6101 |
| Decision Tree | 0.9473 | 0.5583 | 0.5783 | 0.537 | 0.5869 | 0.4091 | 0.6136 | 0.6028 | 0.6108 |
| K-Nearest Neighbors | 0.9562 | 0.645 | 0.5449 | 0.5213 | 0.5409 | 0.3584 | 0.5992 | 0.5944 | 0.5929 |

Units and Definitions: F1-Score combines precision and recall; values closer to 1 are better.

Table showing the F1-scores for classes 0–5, accuracy, macro-average, and weighted-average scores of various models. Models are sorted by Macro Avg F1-Score.
