# Supplementary material for: Single-cell signaling network profiling during redox stress reveals dynamic redox regulation in immune cells
Source: Nat Commun. 2025 Jul 1;16:5600. doi: 10.1038/s41467-025-60727-z (PMC12215076; doi:10.1038/s41467-025-60727-z)
Supplement: Supplementary file 2 — Reporting Summary [file 41467_2025_60727_MOESM2_ESM.pdf]

nature portfolio

Corresponding author(s): Shih-Yu Chen

Last updated by author(s): May 12, 2025

Reporting Summary

Nature Portfolio wishes to improve the reproducibility of the work that we publish. This form provides structure for consistency and transparency in reporting. For further information on Nature Portfolio policies, see our Editorial Policies and the Editorial Policy Checklist.

Statistics

For all statistical analyses, confirm that the following items are present in the figure legend, table legend, main text, or Methods section.

n/a	Confirmed	
☐	☒	The exact sample size (*n*) for each experimental group/condition, given as a discrete number and unit of measurement
☐	☒	A statement on whether measurements were taken from distinct samples or whether the same sample was measured repeatedly
☐	☒	The statistical test(s) used AND whether they are one- or two-sided<br>*Only common tests should be described solely by name; describe more complex techniques in the Methods section.*
☒	☐	A description of all covariates tested
☐	☒	A description of any assumptions or corrections, such as tests of normality and adjustment for multiple comparisons
☐	☒	A full description of the statistical parameters including central tendency (e.g. means) or other basic estimates (e.g. regression coefficient) AND variation (e.g. standard deviation) or associated estimates of uncertainty (e.g. confidence intervals)
☐	☒	For null hypothesis testing, the test statistic (e.g. *F*, *t*, *r*) with confidence intervals, effect sizes, degrees of freedom and *P* value noted<br>*Give P values as exact values whenever suitable.*
☒	☐	For Bayesian analysis, information on the choice of priors and Markov chain Monte Carlo settings
☒	☐	For hierarchical and complex designs, identification of the appropriate level for tests and full reporting of outcomes
☐	☒	Estimates of effect sizes (e.g. Cohen's *d*, Pearson's *r*), indicating how they were calculated

Our web collection on statistics for biologists contains articles on many of the points above.

Software and code

Policy information about availability of computer code

Data collection	Mass cytometry acquired on a CyTOF2 (Fluidigm)<br>Flow cytometry aquistion was performed by LSRII cytometer (BD Biosciences)
Data analysis	The following software and R packages were used for data analysis:<br><br>Cytobank.org<br><br>R version<br>R 4.3.1 binary for macOS 11 (Big Sur)<br>SCORPIUS 1.0.8<br>Seurat Version 4.2.0<br>dplyr 1.1.4<br>ggplot2 3.4.2<br>pheatmap Version: 1.0<br>viridis 0.6.3<br>scales 1.3.0<br>RColorBrewer 1.1-3<br>patchwork 1.1.3

Python 3.9.5
scikit-learn 1.2.1
xgboost 1.6.2
Catboost 1.1

FlowJo(Version 10)
GraphPad PRISM (Version 7)
MATLAB Online Versions for analyzed DREVI plot
Morpheus software for heatmap plotting

For manuscripts utilizing custom algorithms or software that are central to the research but not yet described in published literature, software must be made available to editors and reviewers. We strongly encourage code deposition in a community repository (e.g. GitHub). See the Nature Portfolio guidelines for submitting code & software for further information.

Data

Policy information about availability of data

All manuscripts must include a data availability statement. This statement should provide the following information, where applicable:

- Accession codes, unique identifiers, or web links for publicly available datasets
- A description of any restrictions on data availability
- For clinical datasets or third party data, please ensure that the statement adheres to our policy

Single-cell mass cytometry (CyTOF) datasets for ROS analysis of human whole blood populations, OT-I CD8 mice in vitro T cell activation(hypoxia/ normoxia and N-AC treatment), MC38 B6 mice CD8T cells, LCMV mouse CD8 T cells, CAR-T patients, Hepatocellular carcinoma patients, and hemodialysis patients analysis of ROS states, and flow cytometry data for flourescence cell barcoding to screen 6 immune cell types with different conditional and concentrations of hydrogen peroxide treatment are publicly available at Zenodo via the DOI 10.5281/zenodo.11541294.

Research involving human participants, their data, or biological material

Policy information about studies with human participants or human data. See also policy information about sex, gender (identity/presentation), and sexual orientation and race, ethnicity and racism.

Reporting on sex and gender	All the following human samples were Asian and detailed information described in Supplementary Table 4.<br>For the 10 healthy donor samples contain with 5 males and 5 females (1 of the females is European).<br>CAR-T patients are 3 males and 4 females.<br>Hepatocellular Carcinoma patients were both 2 males.<br>And hemodialysis patients were including 15 males and 18 females together with 3 males and 3 females as healthy donors.
Reporting on race, ethnicity, or other socially relevant groupings	All the following human samples were Asian and detailed information provided on Table 4.<br>Only the 10 healthy donor samples contain including 1 female who is European.<br>Importantly, this study did not involve any diagnosis specific to age, gender, or race, or draw any conclusions based on these patients characteristics.
Population characteristics	All human sample donors are described in Supplementary Table 4, 8, 11, and 14.
Recruitment	All patient-related protocols were reviewed and approved by the Institutional Review Board (IRB) at the corresponding medical institutes. Samples were collected after obtaining informed consent.
Ethics oversight	Hepatocellular carcinoma patient samples and samples from healthy donors for the lineage analyses were recruited at the National Taiwan University Hospital, Taipei, Taiwan (IRB No. 201912040RINA). Hemodialysis patients were recruited at Kaohsiung Medical University Hospital (IRB No. KMUHIRB-E(I)-20200109). CAR-T patient samples were collected at the National Taiwan University Cancer Center as part of two interventional clinical trials registered at ClinicalTrials.gov (NCT04943016 and NCT03624686). The studies were approved by the Institutional Review Board of the National Taiwan University Hospital (IRB No. 202103150MIPC and 201711021RIND), and written informed consent was obtained from all participants.

Note that full information on the approval of the study protocol must also be provided in the manuscript.

Field-specific reporting

Please select the one below that is the best fit for your research. If you are not sure, read the appropriate sections before making your selection.

- [x] Life sciences
- [ ] Behavioural & social sciences
- [ ] Ecological, evolutionary & environmental sciences

For a reference copy of the document with all sections, see nature.com/documents/nr-reporting-summary-flat.pdf

Life sciences study design

All studies must disclose on these points even when the disclosure is negative.

Sample size	Sample sizes were maximized based on availability of clinical samples.<br>With the OT-I mouse model, LCMV mouse model, and MC38 injection to B6 mice, we had three mice for each time experiment.
Data exclusions	No samples were excluded.
Replication	For human whole blood lineage-specific ROS profiles were replicated by repeating the experiments.<br>And all the OT-I mice, LCMV mouse model, and MC38 B6 mice experiments were triplicate, except hypoxia/ normoxia experiment had only twice.
Randomization	Due to the large number of hemodialysis patients, they needed to be divided into two groups for CyTOF operation. This division was based on their patient numbers, ordered from smallest to largest.
Blinding	This study did not involve an intervention with control and test groups, therefore, blinding was not possible during analysis.

Reporting for specific materials, systems and methods

We require information from authors about some types of materials, experimental systems and methods used in many studies. Here, indicate whether each material, system or method listed is relevant to your study. If you are not sure if a list item applies to your research, read the appropriate section before selecting a response.

Materials & experimental systems

n/a	Involved in the study	
☐	☒	Antibodies
☐	☒	Eukaryotic cell lines
☒	☐	Palaeontology and archaeology
☐	☒	Animals and other organisms
☐	☒	Clinical data
☒	☐	Dual use research of concern
☒	☐	Plants

Methods

n/a	Involved in the study	
☒	☐	ChIP-seq
☐	☒	Flow cytometry
☒	☐	MRI-based neuroimaging

Antibodies

Antibodies used	All antibodies (over 103) tested and used in the experiments for this study are detailed in Supplementary Table 1.
Validation	To validate ROS-related antibodies for the SN-ROP panel, 103 antibodies were screened across various conditions combining different hydrogen peroxide concentrations (0, 0.1, or 1 μg/ml; and 0, 0.01, 0.07, or 1 μg/ml for Alexa Fluor 700) and stimulation time points. Cells were stained using amine-reactive fluorescent dyes (Pacific Orange, DyLight 350, Alexa Fluor 546, and Alexa Fluor 700) and detected with Alexa Fluor 488-conjugated secondary antibodies. Data were acquired using an LSRII HTS cytometer (BD Biosciences) and analyzed with FlowJo software. ASINH-transformed mean fluorescence intensities (MFIs) were calculated at 0 hours and averaged across three independent replicates to define a baseline for each antibody. Antibodies showing more than a 10% deviation from this baseline under any treatment condition (72 out of 103) were selected for further analysis. A correlation matrix was constructed to evaluate co-regulation patterns among these antibodies, and they were grouped into seven modules. Within each module, antibodies were ranked based on a weighted average score reflecting their dynamic responsiveness, and representative features were selected to maintain balanced module representation. All antibodies were stained at a 1:100 dilution.

Eukaryotic cell lines

Policy information about cell lines and Sex and Gender in Research

Cell line source(s)	RAW264.7, SM826, SH-SY5Y,HUVEC, HL-1, and Jurkat
Authentication	No authentication was performed.
Mycoplasma contamination	No mycoplasma contamination had been detected.
Commonly misidentified lines<br>(See ICLAC register)	No cell lines were misidentified according to the ICLAC register.

Animals and other research organisms

Policy information about studies involving animals; ARRIVE guidelines recommended for reporting animal research, and Sex and Gender in Research

Laboratory animals	OT-I, B6 mice
Wild animals	OT-1 mice (C57BL/6-Tg(TcraTcrb)1100Mjb/J) were purchased from The Jackson Laboratory (Jax 003831).<br>B6 mice (C57BL/6JNarl) were purchased from the Taiwan National Laboratory Animal Center (RMRC 11109).
Reporting on sex	All mice used in this study were male and 8–10 weeks old at the time of experiment.
Field-collected samples	The mice were housed in specific pathogen-free facilities at the Academia Sinica SPF Animal Facility.
Ethics oversight	The protocols for the mouse experiments were approved by the Institutional Animal Care and Use Committee of Academia Sinica (Protocol No. 19-01-1279).

Note that full information on the approval of the study protocol must also be provided in the manuscript.

Clinical data

Policy information about clinical studies
All manuscripts should comply with the ICMJEguidelines for publication of clinical research and a completedCONSORT checklist must be included with all submissions.

Clinical trial registration	NCT04943016 and NCT03624686 (both registered on ClinicalTrials.gov).
Study protocol	The full trial protocols are not publicly available online. However, both studies were conducted under IRB-approved protocols at National Taiwan University Cancer Center (IRB Nos. 202103150MIPC and 201711021RIND).
Data collection	CAR-T patient blood samples were collected at National Taiwan University Cancer Center as part of two clinical trials (NCT04943016 and NCT03624686). Sample collection followed IRB-approved protocols. Samples were obtained at specified time points post-infusion, processed, and cryopreserved before analysis.
Outcomes	This study analyzed immune signaling and redox status in CAR-T patient samples as exploratory endpoints. These outcomes were not part of the primary or secondary endpoints of the original trials but were assessed post hoc to evaluate immune state and oxidative signaling using the SN-ROP platform.

Plants

Seed stocks	*Report on the source of all seed stocks or other plant material used. If applicable, state the seed stock centre and catalogue number. If plant specimens were collected from the field, describe the collection location, date and sampling procedures.*
Novel plant genotypes	*Describe the methods by which all novel plant genotypes were produced. This includes those generated by transgenic approaches, gene editing, chemical/radiation-based mutagenesis and hybridization. For transgenic lines, describe the transformation method, the number of independent lines analyzed and the generation upon which experiments were performed. For gene-edited lines, describe the editor used, the endogenous sequence targeted for editing, the targeting guide RNA sequence (if applicable) and how the editor was applied.*
Authentication	*Describe any authentication procedures for each seed stock used or novel genotype generated. Describe any experiments used to assess the effect of a mutation and, where applicable, how potential secondary effects (e.g. second site T-DNA insertions, mosiacism, off-target gene editing) were examined.*

Flow Cytometry

Plots

Confirm that:

- [x] The axis labels state the marker and fluorochrome used (e.g. CD4-FITC).
- [x] The axis scales are clearly visible. Include numbers along axes only for bottom left plot of group (a 'group' is an analysis of identical markers).
- [x] All plots are contour plots with outliers or pseudocolor plots.
- [x] A numerical value for number of cells or percentage (with statistics) is provided.

Methodology

Sample preparation	Cells were incubated at 37 °C in a 5% CO_2 environment. Subsequently, the cells were stimulated with H_2O_2 at concentrations of 0, 10, and 100 µM for 0, 0.5, 4, and 48 hours. After stimulation, cells were immediately resuspended and washed with PBS.

	And stained with LIVE/DEAD™ Fixable Violet Dead Cell Stain (Invitrogen™) diluted 1:1000 in PBS, and the staining was performed for 30 minutes at room temperature. The cells were then washed once with PBS and fixed using 1.6% paraformaldehyde (PFA; Electron Microscopy Sciences) in PBS for 10 minutes at room temperature. After fixation, the cells were washed and stored in 100-µl aliquots in a 1:10 solution of DMSO (Sigma) in PBS and stored in -80C for scheduled flow cytometry staining.
Instrument	All flow cytometry data was collected on a BD LSRII instrument.
Software	All flow cytometry data was analyzed by FlowJo (Version 10)
Cell population abundance	No cell populations were sorted.
Gating strategy	By using SSC-A vs, FSC-A plot to gate the lymphocytes. Then, the single cells were gated using the FSC-H vs. FSC-A plot, and live cells were gated by Live/dead™ Fixable Violet Dead Cell Stain negative.

☒ Tick this box to confirm that a figure exemplifying the gating strategy is provided in the Supplementary Information.
